# Dietary sulfur amino acid restriction in humans with overweight and obesity: Evidence of an altered plasma and urine sulfurome, and a novel metabolic signature that correlates with loss of fat mass and adipose tissue gene expression

**DOI:** 10.1016/j.redox.2024.103192

**Published:** 2024-05-17

**Authors:** Thomas Olsen, Kathrine J. Vinknes, Kristýna Barvíková, Emma Stolt, Sindre Lee-Ødegård, Hannibal Troensegaard, Hanna Johannessen, Amany Elshorbagy, Jitka Sokolová, Jakub Krijt, Michaela Křížková, Tamás Ditrói, Péter Nagy, Bente Øvrebø, Helga Refsum, Magne Thoresen, Kjetil Retterstøl, Viktor Kožich

**Affiliations:** a Department of Nutrition, Institute of Basic Medical Sciences, Faculty of Medicine University of Oslo, Postboks 1046 Blindern, 0317 Oslo, Norway; b Department of Pediatrics and Inherited Metabolic Disorders, Charles University, First Faculty of Medicine, and General University Hospital, Ke Karlovu 2, 128 00 Prague, Czech Republic; c Department of Endocrinology, Morbid Obesity and Preventive Medicine, Institute of Clinical Medicine, Faculty of Medicine, University of Oslo, Postboks 4959 Nydalen, OUS HF Aker sykehus, 0424 Oslo, Norway; d Department of Pathology, Oslo University Hospital, Rikshospitalet, Postboks 45980 Nydalen, OUS HF Rikshospitalet, 0424 Oslo, Norway; e Department of Physiology, Faculty of Medicine, University of Alexandria, Chamblion street, Qesm Al Attarin, Alexandria 5372066, Egypt; f Department of Pharmacology, University of Oxford, Mansfield Rd, Oxford OX1 3QT, UK; g Department of Molecular Immunology and Toxicology and the National Tumor Biology Laboratory, National Institute of Oncology, Ráth György u. 7-9, 1122 Budapest, Hungary; h Department of Anatomy and Histology, HUN-REN–UVMB Laboratory of Redox Biology Research Group, University of Veterinary Medicine, 1078 Budapest, Hungary; i Chemistry Institute, University of Debrecen, 4012 Debrecen, Hungary; j Department of Food Safety, Norwegian Institute of Public Health, Postboks 222 Skøyen, 0213 Oslo, Norway; k Department of Biostatistics, Institute of Basic Medical Sciences, University of Oslo, Postboks 1122 Blindern, 0317 Oslo, Norway; l The Lipid Clinic, Department of Endocrinology, Morbid Obesity and Preventive Medicine, Oslo University Hospital, Postboks 4959 Nydalen, OUS HF Aker sykehus, 0424 Oslo, Norway

**Keywords:** Sulfur amino acid restriction, Methionine, Cysteine, Transsulfuration, Hydrogen sulfide, Adipose tissue, Gene expression, Randomized controlled trial

## Abstract

**Background:**

In animals, dietary sulfur amino acid restriction (SAAR) improves metabolic health, possibly mediated by altering sulfur amino acid metabolism and enhanced anti-obesogenic processes in adipose tissue.

**Aim:**

To assess the effects of SAAR over time on the plasma and urine SAA-related metabolites (sulfurome) in humans with overweight and obesity, and explore whether such changes were associated with body weight, body fat and adipose tissue gene expression.

**Methods:**

Fifty-nine subjects were randomly allocated to SAAR (∼2 g SAA, n = 31) or a control diet (∼5.6 g SAA, n = 28) consisting of plant-based whole-foods and supplemented with capsules to titrate contents of SAA. Sulfurome metabolites in plasma and urine at baseline, 4 and 8 weeks were measured using HPLC and LC-MS/MS. mRNA-sequencing of subcutaneous white adipose tissue (scWAT) was performed to assess changes in gene expression. Data were analyzed with mixed model regression. Principal component analyses (PCA) were performed on the sulfurome data to identify potential signatures characterizing the response to SAAR.

**Results:**

SAAR led to marked decrease of the main urinary excretion product sulfate (p < 0.001) and plasma and/or 24-h urine concentrations of cystathionine, sulfite, thiosulfate, H_2_S, hypotaurine and taurine. PCA revealed a distinct metabolic signature related to decreased transsulfuration and H_2_S catabolism that predicted greater weight loss and android fat mass loss in SAAR vs. controls (all p_interaction_ < 0.05). This signature correlated positively with scWAT expression of genes in the tricarboxylic acid cycle, electron transport and β-oxidation (FDR = 0.02).

**Conclusion:**

SAAR leads to distinct alterations of the plasma and urine sulfurome in humans, and predicted increased loss of weight and android fat mass, and adipose tissue lipolytic gene expression in scWAT. Our data suggest that SAA are linked to obesogenic processes and that SAAR may be useful for obesity and related disorders.

**Trial identifier:**

https://clinicaltrials.gov/study/NCT04701346.

## Highlights

• Dietary sulfur amino acid restriction alters the sulfurome, including H_2_S, in humans.
• Sulfur-saving strategies were indicated by decreased cysteine catabolism.
• A distinct metabolic sulfurome signature was identified that predicted fat mass loss.
• The metabolic signature further correlated with adipose tissue β-oxidation.

## Graphical abstract

**Figure:**
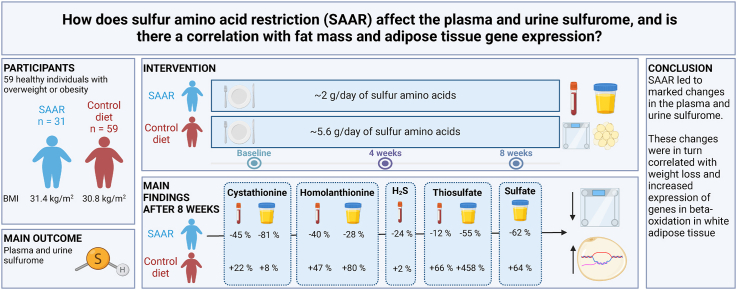

## Nomenclature

AdoHcy: S-adenosylhomocysteine
AdoMet: S-adenosylmethionine
Bet: betaine
BMI: body mass index
CBS: cystathionine ß-synthase
CDO1: cysteine dioxygenase 1
Chol: choline
Csth: cystathionine
CTH: cystathionine γ-lyase
Cys: cysteine
DMG: dimethylglycine
EAR: estimated average requirements
EF: excretional fraction
EMM: estimated marginal means
FAO/WHO: Food and Agriculture Organization/World Health Organization
Gly: glycine
gSD: geometric standard deviation
GSH: glutathione
Hcy: homocysteine
Hlan: homolanthionine
HPLC: high-performance liquid chromatography
HpT: hypotaurine
H2S: hydrogen sulfide
Lan: lanthionine
Met: methionine
NHANES: National Health and Nutrition Examination Survey
PC: principal component
PCA: principal component analysis
PPARγ: peroxisome proliferator-activated receptor γ
RDA: recommended daily allowance
SAA: sulfur amino acids
SAAR: sulfur amino acid restriction
Ser: serine
SO32-: sulfite
SO42-: sulfate
S2O32-: thiosulfate;
SSC: S-sulfocysteine
Tau: taurine
tCys: total cysteine
tCysGly: total cysteinylglycine
tGGCys: total γ-glutamylcysteine
tGSH: total glutathione
tHcy: total homocysteine
USDA: United States Department of Agriculture

## 1 Introduction

**Fig. 1:**
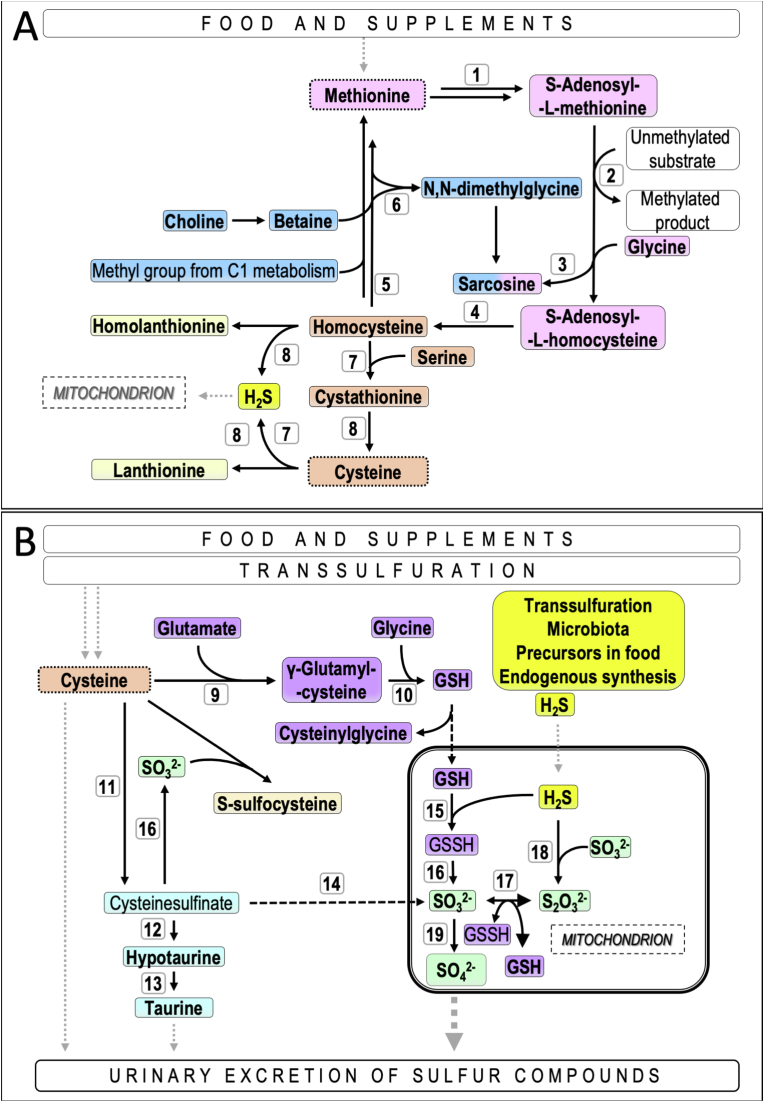
**Panel A.** Methionine cycle (pink), homocysteine remethylation (blue), transsulfuration (orange) and desulfhydration (light yellow). Methionine is converted by methionine adenosyltransferases (**1**, MAT I/III and MAT II) to S-adenosylmethionine (AdoMet). The methyl group of AdoMet is used in methylation reactions catalyzed by methyl transferases (**2**), yielding S-adenosylhomocysteine (AdoHcy); excess methyl groups are removed by glycine N-methyltransferase (**3**), yielding sarcosine. AdoHcy is cleaved by S-adenosylhomocysteine hydrolase (**4**) to homocysteine and adenosine. Homocysteine can be remethylated to methionine by the folate/vitamin B_12_-dependent methionine synthase reaction (**5**) or by the betaine-homocysteine methyltransferase reaction (**6**). Alternatively, homocysteine can enter the transsulfuration pathway. Homocysteine is condensed with serine in a reaction catalyzed by cystathionine β-synthase (CBS, **7**) to form cystathionine, which is subsequently cleaved to cysteine, α-ketobutyrate and ammonia by cystathionine γ-lyase (CTH, **8**). CBS (**7**) preferentially utilizes a combination of cysteine and homocysteine to synthesize the gasotransmitter hydrogen sulfide (H_2_S) [69]and CTH (**8**) is capable of producing H_2_S also by condensing two Hcy molecules; lanthionine and homolanthionine are byproducts of these H_2_S- synthesizing reactions. Cysteine is a precursor for glutathione synthesis catalyzed by glutamate cysteine ligase (**9)** and glutathione synthase **(10**), as well as for taurine synthesis. The reactions in taurine synthesis are catalyzed by cysteine dioxygenase 1 (**11)** and cysteine sulfinate decarboxylase (**12)**, followed by conversion of hypotaurine to taurine by flavin-containing monooxygenase 1 (**13**). Cysteinesulfinate can be also a precursor of sulfite formation by the cytosolic aspartate aminotransferase (AST) (**14**) (Panel B). **Panel B.** Metabolism of glutathione (purple) and taurine (cyan blue), and synthesis and catabolism of hydrogen sulfide (yellow and green). H_2_S condenses with reduced glutathione to form glutathione persulfide in a reaction catalyzed by sulfide:quinone oxidoreductase (**15**), followed by oxidation to sulfite by persulfide dioxygenase (PDO) (**16**) and interconversions between sulfite and thiosulfate involving PDO (**17**) and thiosulfate transferase (**18**). In the final step, sulfite is oxidized to sulfate by sulfite oxidase containing the molybdenum cofactor (**19**). Sulfite can alternatively react non-enzymatically with Cys forming S-sulfocysteine (light orange). Abbreviations: AdoMet, S-adenosylmethionine; AdoHcy, S-adenosylhomocysteine; GSH, glutathione; GSSH, glutathione persulfide; H_2_S, free sulfide; SO_3_^2−^, sulfite; S_2_O_3_^2−^; thiosulfate; SO_4_^2−^, sulfate. Analytes in bold were measured and are reported in this study. (For interpretation of the references to colour in this figure legend, the reader is referred to the Web version of this article.)

Methionine (Met) is an essential amino acid while cysteine (Cys) is semi-essential and conditional on adequate Met intake [1]. Metabolism of these two sulfur amino acids (SAA) provides numerous intermediates with important biological functions, such as the universal methyl group donor S-adenosylmethionine (AdoMet), the signaling molecule hydrogen sulfide (H_2_S), the major redox regulator glutathione (GSH) and the osmolyte taurine (Tau). The proximal part of SAA metabolism encompasses the methionine cycle, homocysteine remethylation and transsulfuration (including the related one-carbon metabolism); for details see Fig. 1A. The distal part of SAA metabolism includes Cys catabolism leading to the synthesis and metabolism of H_2_S, GSH and Tau (Fig. 1B).

According to the FAO/WHO [2], the estimated average requirements (EAR) of total SAA intake in adults is 15 mg/kg body weight/day (10.5 mg/kg body weight for Met, 4.1 mg/kg body weight for Cys). The recommended daily allowance (RDA) provided by the USDA is 19 mg/kg body weight for total SAA [3]. Recent publications from large US cohorts report that most individuals significantly exceed the EAR and RDA [[4], [5], [6], [7]], whereas intake recently reported in a Dutch population was somewhat lower [8]. In the NHANES population, average intakes in upper intake quantiles were reported to be > 60 mg/kg/body weight [5,7], similar to the Framingham and Framingham Offspring cohorts [6]. Such high intakes have been positively associated with diabetes risk [6] and related mortality [4], and cardiovascular disease risk scores [5]. Furthermore, observational studies have reported that high plasma total cysteine (tCys) was positively and strongly associated to obesity and fat mass [[9], [10], [11], [12], [13]], and impaired glucose metabolism [14,15], whereas high plasma Met was positively associated with liver fat [13]. Animal studies have demonstrated that dietary sulfur amino acid restriction (SAAR) can prolong life [16], improve body composition and insulin sensitivity [17], prevent fatty liver [18] and cognitive impairment [19], and influence tumor cell metabolism [20]. The potential underlying mechanisms appear to involve altered sulfur metabolism as demonstrated in animal knockout models [21], altered H_2_S metabolism [22,23], and distinct effects of Cys on adipogenic gene expression [24]. Notably, H_2_S levels appear to be essential for dietary restriction benefits, where dietary SAA add-back abrogates such effects [25]. In contrast, increased H_2_S production parallels accelerated weight gain in *Cdo1*^−/−^ mice that accumulate Cys [26,27]. Surprisingly, increased Cys intake did not induce obesity while it ameliorated insulin resistance in the SHR-CRP rats, possibly due to beneficial effects of accumulating taurine (Tau) [28]. Thus, the significance of SAA metabolism in relation to metabolic health appears to be pivotal, necessitating further in-depth investigation, especially within the context of human physiology.

In a recently published double-blind randomized controlled dietary intervention study, we showed that dietary SAAR can have beneficial metabolic effects when applied in humans with overweight and obesity [29]. However, the impact of SAAR on endogenous sulfur compounds and related metabolites (termed sulfurome) have not been examined in humans, and it is not known whether such potential effects are associated with the change in metabolic phenotypes. Thus, the overreaching aim of the current study was to advance our understanding of how dietary SAAR influence the plasma and urinary sulfurome and whether such changes were associated with the improvement of other metabolic phenotypes. Specifically, we performed a comprehensive and targeted investigation of the effect of dietary SAAR on the plasma and urinary sulfurome as well as sulfur balance in humans. Furthermore, we performed principal component analyses (PCA) to detect a sulfur metabolomic signature in response to SAAR, and investigated its association with metabolic phenotypes, and gene expression patterns in scWAT obtained by bioinformatic analyses.

## 2 Methods

### 2.1 Study design and subjects

The study protocol outlining the study design, sample size determination, recruitment strategy, randomization, blinding and dietary intervention can be accessed elsewhere [29,30]. Briefly, 59 healthy, free-living men (n = 16) and women (n = 43) aged between 18 and 45 with overweight or obesity (body mass index [BMI] 27 to 35) were stratified by sex and randomized with the aim to achieve equal sized groups. Participants received a diet either low (1.9 g/day for men and 1.8 g/day for women, SAAR group) or high (5.5 g/day for men and 5.4 g/day for women, control group) in SAA for 8 weeks. Data collection occurred at baseline, week 4 and week 8. The base diet was identical in both groups and the mean energy content was 2401 kcal/day for men and 2030 kcal/day for women. The base diet provided 34 energy % (E%) from fat, 46 E% from carbohydrate and 13 E% from protein, which is in line with the Nordic nutrition recommendations [31]. Taking subject body weight at baseline into consideration, average SAA and protein content in the base diet was composed of natural foods (∼19.9 mg/kg/day SAA and ∼0.62 g/kg/day of protein), and Met- and Cys-free amino acid supplements (∼0.16 g/kg/day of protein). Subjects were allowed one *ad libitum* meal per week which brought the total SAA and protein in the base diet + amino acid supplement to ∼22.2 mg/kg/day and ∼0.86 g/kg/, respectively. Dietary SAA was further titrated in the control group using capsules containing methionine and cysteine (3.6 g/day) whereas the SAAR group received capsules containing maltodextrin (3 g/day). The control group thus had average SAA intakes at 65 mg/kg/day, whereas the SAAR group was in line with the base diet (∼22.2 mg/kg/day).

Participants and study personnel were blinded to the intervention. All participants gave informed consent, and the study was conducted according to the Declaration of Helsinki. The study was approved by the Regional Ethical Committee South-East (Ref no. 123644).

### 2.2 Blood and urine sampling

For SAA assays, blood was collected into a 5 mL lithium-heparin gel tube, which was immediately placed in an ice bath, and centrifuged within 15 min at 2000*g* at 4 °C for 5 min. Separated plasma samples were immediately frozen at −80 °C and stored at −80 °C for up to 3 months prior to analysis.

Twenty-four-hour urine collection was performed prior to baseline after the participants had voided in the morning. All urine after this point was collected in 3 L containers with light protection and stored at 4 °C during collection. Samples were subsequently aliquoted and stored at −80 °C for up to 3 months prior to analysis.

### 2.3 Outcomes

The main outcomes of this work included the sulfur-containing metabolites and related compounds. Analytes (and their abbreviations) that were determined in plasma and 24-h urine, and calculated excretional fraction (EF) in the appropriate metabolic pathway are shown in Table 1. We determined all analytes except SO_4_^2−^ in plasma, and γ-glutamylcysteine (tGGCys) and hypotaurine (HpT) in urine. Due to matrix interferences, SO_4_^2−^ was not measured in plasma. Concentrations of tGGcys and HpT in urine were below the lower limit of quantification. Due to matrix interferences, sulfite (SO_3_^2−^) was measured in 47 plasma samples and in 26 urine samples only. EFs were calculated for analytes that were determined in both urine and plasma except for total aminothiols as the major fraction (i.e., protein-bound) of aminothiols is not freely filtered in glomeruli.

**Table 1:** Overview of outcomes.

Analyte	Abbreviation	Plasma	Urine	EF^b^
Methionine cycle and transsulfuration				
Methionine	Met	YES	YES	YES
S-adenosylmethionine	AdoMet	YES	n.d.	N/A
Sarcosine (N-methylglycine)	Sarcosine	YES	YES	YES
S-adenosylhomocysteine	AdoHcy	YES	n.d.	N/A
Total homocysteine	tHcy	YES	YES	N/A
Cystathionine	Csth	YES	YES	YES
Total cysteine	tCys	YES	YES	N/A
***Homocysteine remethylation and one-carbon metabolism***				
Choline	Chol	YES	YES	YES
Betaine (N,N,N-trimethylglycine)	Bet	YES	YES	YES
N,N-dimethylglycine	DMG	YES	YES	YES
Glycine	Gly	YES	YES	YES
Serine	Ser	YES	YES	YES
***Glutathione-related metabolites***				
Total glutathione	tGSH	YES	YES	N/A
Total cysteinylglycine	tCysGly	YES	YES	N/A
Total γ-glutamylcysteine	tGGCys	YES	<LLOQ	N/A
***Taurine-related metabolites***				
Hypotaurine	HpT	YES	<LLOQ	N/A
Taurine	Tau	YES	YES	YES
***Hydrogen sulfide synthesis and catabolism***				
Sulfide, bioavailable	H_2_S	YES	n.d.	N/A
Homolanthionine	Hlan	YES	YES	YES
Lanthionine	Lan	YES	YES	YES
Sulfite^a^	SO_3_^2-^	YES	YES	YES
S-sulfocysteine	SSC	YES	YES	YES
Thiosulfate	S_2_O_3_^2-^	YES	YES	YES
Sulfate	SO_4_^2-^	n.d.	YES	N/A

n.d., not determined (analysis not performed).

N/A, not applicable.

<LLOQ, analyte concentration below the lower limit of quantification.

a analyte determined in n = 47 with plasma and n = 26 with 24-h urine.

b EF cannot be calculated for total aminothiols as these are not freely filtered in glomeruli.

### 2.4 Biochemical analyses

Sulfur metabolites and related compounds were determined by chromatographic methods. Briefly, reversed phase HPLC separation of compounds fluorescently labelled with monobromobimane was used for analysis of SO_3_^2−^, thiosulfate (S_2_O_3_^2−^) and bioavailable sulfide (H_2_S) as described previously [28,32]. Aminothiols – total homocysteine (tHcy), tCys, total glutathione (tGSH), total cysteinylglycine (tCysGly) and total γ-glutamylcysteine (tGGCys) were determined after reduction of disulfides and plasma protein bound fractions with tris(2-carboxyethyl)phosphine hydrochloride followed by derivation with 7-fluorobenzofurazan-4-sulfonic acid ammonium salt and separated by reversed phase HPLC with fluorescence detection [33]. Other amino acids and related metabolites including thioethers cystathionine, lanthionine (Lan) and homolanthione (Hlan) were determined by LC-MS/MS method using commercially available Phenomenex EZ:faast kit for amino acid analysis as described previously [34]. Choline (Chol), betaine (Bet), dimethylglycine (DMG), hypotaurine (HpT), taurine (Tau) and S-sulfocysteine (SSC) were determined by the LC-MS/MS method described in detail elsewhere [28,32]. SAM and SAH levels in plasma and tissue extracts were determined based on chromatographic separation on a Hypercarb column filled with porous graphitic carbon stationary phase using the previously published method [35]. Urinary SO_4_^2−^was determined by a newly developed method using ion-exchange chromatography with conductivity detection as described elsewhere [29].

### 2.5 Assessment of renal handling of metabolites

The excretional fraction of metabolites is a measure of renal reabsorption/secretion of the metabolite. It was calculated using the standard formula: EF_metabolite_ [%] = 100 x (U_metabolite_ x P_creatinine_)/(P_metabolite_ x U_creatinine_), where U and P are concentrations of analytes determined in simultaneously obtained urine and plasma samples, respectively. The premise for calculating EF is not fulfilled for plasma total aminothiols as only the small non-protein bound fraction is filtered in glomeruli and EF for these metabolites cannot be estimated. Creatinine concentration in urine and plasma was determined by enzymatic methods.

### 2.6 Estimates of sulfur balance

To estimate the sulfur balance, the intake of the major sulfur compounds in diet was converted to mmol/day by dividing the mg/day by the molecular weight of the respective metabolite (Met, 149.21; Cys, 121.16; SO_4_^2−^, 96.06). Daily Met and Cys intake were calculated as the sum of dietary intake (an average of male and female dietary intake including the *ad libitum* meal) and Met + Cys intake in capsules. The basal plant-based diet contains negligible amounts of Tau, thus Tau intake was estimated at 0 mmol/day. Intake of sulfate was estimated based on an average intake of 500 mg sulfate from drinking-water, air and food [36].

### 2.7 Adipose tissue biopsies and mRNA sequencing

Methods for adipose tissue biopsies, RNA isolation and mRNA sequencing have been published previously [29]. Briefly, scWAT was obtained from the periumbilical region and immediately dissected and snap frozen in liquid nitrogen, and stored at −80 °C until analysis. RNA was isolated using a modified protocol supplied by the manufacturer (Nucleospin RNA Mini kit for RNA purification, Macherey–Nagel, Germany), and an RNA integrity number of >7 was considered to be of sufficient quality for further analysis (Agilent RNA 6000 Nano Kit, Agilent Technologies, CA, USA). RNA from 51 participants, who had complete sets of scWAT, were sent to the Norwegian Sequencing Centre (www.sequencing.uio.no) for analysis.

### 2.8 Bioinformatics

Gene expression was quantified and normalized using the trimmed mean of M-values algorithm, and calculated as the percent change from baseline to the end of the intervention. We then ran one linear regression model for each mRNA with percent change in mRNA as a function of principal component 1 (PC1, see method below) by group interaction. The p-values were corrected for multiple testing using the Benjamini-Hochberg approach [37]. In addition to the interaction term, the model contained PC1 and group as separate independent variables. The top 500 mRNAs (all with corrected p < 0.05 for the interaction term) were then subjected to pathway analysis using hypergeometric tests against the msigDB canonical pathways data base. Identification of key drivers were done using the Mergeomics data base using the top 1000 mRNAs (all with corrected p < 0.05). To visualize the correlation between PC1 and the percent change in mRNAs in the tricarboxylic acid cycle (TCA) and electron transport chain (ETC) pathway, we calculated the mean expression for all the mRNAs overlapping this pathway.

### 2.9 Statistical analyses

Baseline characteristics are presented as mean (standard deviations). As most metabolites were highly skewed at baseline, data were log-transformed before analysis and presented as geometric means (gMean) (geometric standard deviation [gSD]) [38]. Outcome analyses were performed using baseline-adjusted linear mixed model regression with the log-transformed metabolite as the dependent variable and grouping (SAAR, control) variable, visit variables (4 weeks and 8 weeks), and their interaction terms. The model was further adjusted for sex (stratification variable). A random intercept for subject ID was added to the model to account for within-subject correlation. Adjustment for baseline differences was performed as recommended by Twisk [39]. Obtained estimates and 95 % confidence intervals (CI) were back-transformed and represent % change vs. controls. Estimated marginal (predicted) geometric means derived from the models can be found in the online appendix. All subjects were included in the final analysis (intention-to-treat). To identify sulfurome-related metabolic signatures to the intervention, we performed PCA including the data on 8-week changes in all metabolites measured in plasma and urine. Correlation coefficients between the resulting PC1 and change in metabolic phenotypes including body weight, body fat mass compartments and gene expression data were calculated as Spearman's rho. Regression models assessing the interaction of group and the PCs had body weight/fat mass as the outcome and group, PC1 and their interaction term (group × PC1) as the predictors. Unless otherwise stated, all p-values for the metabolites after 8 weeks were derived from the mixed models. P < 0.05 was considered statistically significant. Benjamini-Hochberg corrected p-values are reported for outcome data of primary interest (8-week metabolite data) [37]. All analyses were performed using R v. 4.2.2.

## 3 Results

**Fig. 2:**
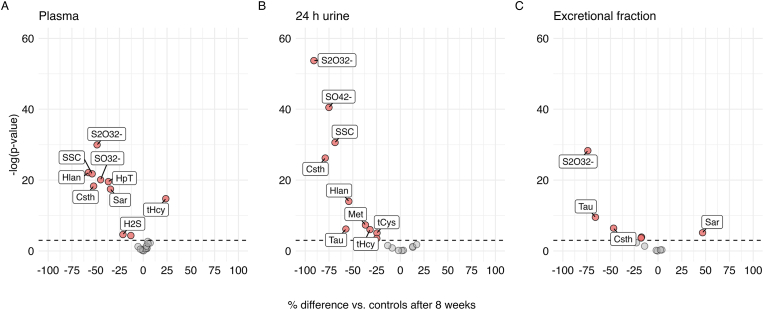
% Difference in A) plasma sulfurome, B) 24 h urine sulfurome and C) excretional fraction of metabolites after 8 weeks in SAAR vs. controls. % change as derived from back-transformed regression estimates derived from a linear mixed model (described in methods); data points above the dashed line indicate p < 0.05. Abbreviations: Chol, choline; Csth, cystathionine; H_2_S, bioavailable sulfide; Hlan, homolanthionine; HpT, hypotaurine; Lan, Lanthionine; Met, methionine; S_2_O_3_^2−^, thiosulfate; SO_4_^2−^, sulfate; SO_3_^2−^, sulfite; Sar, sarcosine; SSC, S-sulfocysteine; Tau, taurine; tCys, total cysteine; tCysGly, total cysteinylglycine; tHcy, total homocysteine.

Baseline characteristics of the study population are presented in Appendix Table A1 in the online appendix. The CONSORT diagram outlining participant flow can be accessed in our previous publication [29]. Briefly, 59 participants were included (SAAR, n = 31, controls, n = 28), with 8 men in each group. Mean (standard deviation) age was 33.2 (5.86) years in the SAAR group and 34.4 (6.26) years in the control group. Despite randomization, participants in the SAAR group had higher body weight at baseline whereas body fat % was similar. Sulfur-containing and related metabolites were similarly distributed across groups at baseline, except for urinary SO_4_^2−^ and Csth both of which were numerically higher in the SAAR group (Table 2). The effects of SAAR on plasma, urine and EF of the sulfur metabolites and related compounds are presented below and in summary figures (Fig. 2A–C). Regression coefficients, 95 % CIs and (adjusted) p-values for between-group comparisons are presented in Table 3, Table 4, Table 5. Unadjusted geometric means and estimated marginal geometric means derived from the statistical models at each timepoint, as well as within-group comparisons can be found in the online appendix (Appendix Tables A.2-A.11).

**Table 2:** Sulfur amino acids and related metabolites in plasma, 24-h urine and excretional fraction^c^ at baseline^a^.

	Plasma, μmol/L		24-h urine, μmol/24 h		Excretional fraction, %	
	SAAR	Controls	SAAR	Controls	SAAR	Controls
Methionine cycle and transsulfuration						
Methionine	21.0 (1.18)	22.4 (1.19)	8.92 (1.86)	7.42 (1.98)	0.24 (1.40)	0.24 (1.45)
S-adenosylmethionine	0.07 (1.19)	0.07 (1.18)	n.d.		N/A	
Sarcosine (N-methylglycine)	1.13 (1.44)	1.22 (1.42)	1.82 (1.97)	1.96 (1.97)	0.74 (1.87)	0.68 (2.03)
S-adenosylhomocysteine	0.02 (1.45)	0.02 (1.30)	n.d.		N/A	
Total homocysteine	9.29 (1.32)	9.43 (1.30)	8.27 (1.67)	6.77 (1.83)	N/A	
Cystathionine	0.23 (1.71)	0.21 (1.49)	36.6 (2.16)	26.5 (2.21)	76.9 (1.83)	74.4 (1.85)
Total cysteine	261 (1.14)	272 (1.16)	243 (1.50)	216 (1.54)	N/A	
***Homocysteine remethylation and one-carbon metabolism***						
Choline	6.03 (1.27)	6.24 (1.25)	24.5 (1.76)	23.3 (1.57)	1.76 (1.42)	1.83 (1.33)
Betaine (N,N,N-trimethylglycine)	25.7 (1.62)	27.1 (1.44)	60.2 (2.12)	55.2 (1.91)	0.94 (2.08)	0.93 (1.73)
N,N-dimethylglycine	3.47 (1.38)	3.51 (1.39)	60.2 (2.26)	53.8 (1.99)	7.87 (2.05)	6.75 (1.80)
Glycine	207 (1.36)	224 (1.35)	1660 (1.84)	1680 (1.68)	3.40 (1.72)	3.46 (1.81)
Serine	98.2 (1.19)	101 (1.24)	405 (1.88)	356 (1.65)	2.04 (1.58)	1.87 (1.55)
***Glutathione-related metabolites***						
Total glutathione	6.35 (1.28)	6.46 (1.28)	6.14 (1.71)	5.59 (1.73)	N/A	
Total cysteinylglycine	25.1 (1.26)	24.9 (1.25)	8.42 (1.58)	7.61 (1.57)	N/A	
Total γ-glutamylcysteine	3.83 (1.22)	4.05 (1.25)	<LLOQ		N/A	
***Taurine-related metabolites***						
Hypotaurine	0.76 (1.4)	0.71 (1.39)	<LLOQ		N/A	
Taurine	56.3 (1.24)	55.8 (1.29)	316 (3.53)	399 (3.48)	2.00 (3.64)	2.83 (2.93)
***Hydrogen sulfide synthesis and catabolism***						
Sulfide, bioavailable	0.05 (1.29)	0.05 (1.30)	n.d.		N/A	
Homolanthionine	0.01 (1.67)	0.01 (1.45)	1.33 (1.70)	1.07 (1.70)	80.0 (1.56)	72.3 (1.42)
Lanthionine	0.05 (1.43)	0.06 (1.23)	14.3 (1.77)	13.2 (1.62)	132 (1.52)	125 (1.39)
Sulfite^b^	0.05 (1.97)	0.05 (2.17)	1.79 (2.7)	1.87 (2.98)	9.99 (2.62)	13.0 (2.00)
S-sulfocysteine	0.14 (1.25)	0.15 (1.22)	6.53 (1.56)	5.72 (1.57)	18.3 (1.49)	17.4 (1.37)
Thiosulfate	0.30 (1.30)	0.31 (1.22)	16.6 (1.71)	13.9 (1.99)	25.7 (1.68)	24.2 (1.45)
Sulfate	n.d.		17100 (1.62)	15700 (1.68)	N/A	

n.d., not determined (analysis not performed).

N/A, not applicable.

<LLOQ, analyte concentration below the lower limit of quantification.

a All values are geometric mean (geometric standard deviations).

b analyte determined in n = 47 with plasma and n = 26 with 24-h urine.

c EF cannot be calculated for total aminothiols as these are not freely filtered in glomeruli.

**Table 3:** β-estimates (95 % and confidence intervals) of plasma sulfur amino acids and related metabolites in sulfur amino acid restricted individuals vs. controls^a^.

	4 weeks		8 weeks		
	β (95 % CI)	p	β (95 % CI)	p	p_adjusted_
***Methionine cycle and transsulfuration***					
Methionine	−2.76 (−10.2, 5.34)	0.49	−5.76 (−12.9, 1.95)	0.14	0.25
S-adenosylmethionine	−0.499 (−5.9, 5.22)	0.86	−1.3 (−6.51, 4.2)	0.63	0.76
Sarcosine (N-methylglycine)	−29.7 (−39.2, −18.8)	<0.001	−34.4 (−43.1, −24.5)	<0.001	<0.001
S-adenosylhomocysteine	1.24 (−12.7, 17.4)	0.87	0.356 (−13, 15.8)	0.96	0.96
Total homocysteine	22.5 (13.1, 32.6)	<0.001	23.7 (14.3, 33.8)	<0.001	<0.001
Cystathionine	−50 (−60.9, −36.1)	<0.001	−52.2 (−62.5, −39.1)	<0.001	<0.001
Total cysteine	5.11 (−0.107, 10.6)	0.055	4.76 (−0.354, 10.1)	0.068	0.15
***Homocysteine remethylation and one-carbon metabolism***					
Choline	−6.73 (−16.1, 3.66)	0.19	−5.43 (−14.8, 4.92)	0.29	0.43
Betaine (N,N,N-trimethylglycine)	−2.31 (−13.1, 9.87)	0.69	3.76 (−7.59, 16.5)	0.53	0.66
N,N-dimethylglycine	−0.21 (−9.41, 9.93)	0.97	3.88 (−5.57, 14.3)	0.43	0.56
Glycine	0.786 (−7.16, 9.41)	0.85	−0.764 (−8.48, 7.6)	0.85	0.9
Serine	4.96 (−2.05, 12.5)	0.17	2.18 (−4.54, 9.38)	0.53	0.66
***Glutathione-related metabolites***					
Total glutathione	−0.0649 (−8.35, 8.97)	0.99	7.3 (−1.47, 16.9)	0.10	0.20
Total cysteinylglycine	3.44 (−2.48, 9.72)	0.26	3.36 (−2.47, 9.55)	0.26	0.40
Total γ-glutamylcysteine	4.69 (−1.85, 11.7)	0.16	5.1 (−1.37, 12)	0.12	0.23
***Cysteine decarboxylation***					
Hypotaurine	−35 (−43.7, −24.9)	<0.001	−36.6 (−45, −26.9)	<0.001	<0.001
Taurine	−1.4 (−12.3, 10.9)	0.81	−3.06 (−13.6, 8.79)	0.60	0.73
***Hydrogen sulfide synthesis and catabolism***					
Sulfide, bioavailable	−15.1 (−29.4, 1.97)	0.079	−21.3 (−34.3, −5.67)	0.01	0.029
Homolanthionine	−49.9 (−61.2, −35.3)	<0.001	−57.7 (−67.1, −45.6)	<0.001	<0.001
Lanthionine	−14.7 (−23.6, −4.66)	0.005	−13 (−22, −2.94)	0.013	0.037
Sulfite	−33.7 (−44.5, −20.9)	<0.001	−44.8 (−53.8, −34.1)	<0.001	<0.001
S-sulfocysteine	−50.2 (−60.2, −37.7)	<0.001	−53.8 (−62.8, −42.7)	<0.001	<0.001
Thiosulfate	−45.1 (−53.4, −35.2)	<0.001	−48.6 (−56.3, −39.6)	<0.001	<0.001

a Values are derived from linear mixed regression models with log-transformed metabolite as the outcome, and group, visit and their interaction term (group × time) at 4 and 8 weeks as predictors. The models were baseline adjusted. Subject ID was added as a random term to account for within-subject correlation. All estimates are back-transformed and represent % difference vs controls. P-values were derived from the regression models. Adjusted 8-week p-value data were calculated using the Benjamini-Hochberg procedure.

**Table 4:** β-estimates (95 % and confidence intervals) of 24-h urine sulfur amino acids and related metabolites in sulfur amino acid restricted individuals vs. controls^a^.

Metabolite	4 weeks		8 weeks		
	β (95 % CI)	p	β (95 % CI)	p	p_adjusted_
***Methionine cycle and transsulfuration***					
Methionine	−43.1 (−55.9, −26.6)	<0.001	−36.7 (−51.2, −18)	<0.001	0.003
Sarcosine (N-methylglycine)	−0.151 (−22.4, 28.5)	0.99	13.1 (−12.5, 46.1)	0.35	0.48
Total homocysteine	−32.8 (−47.3, −14.5)	0.001	−31.9 (−46.8, −12.9)	0.002	0.008
Cystathionine	−77.3 (−84.8, −66)	<0.001	−79 (−86.1, −68.3)	<0.001	<0.001
Total cysteine	−22.1 (−36.1, −5.12)	0.013	−24.6 (−38.3, −7.73)	0.006	0.02
***Homocysteine remethylation and one-carbon metabolism***					
Choline	−16.7 (−33, 3.67)	0.10	−8.63 (−26.9, 14.2)	0.42	0.56
Betaine (N,N,N-trimethylglycine)	−4.6 (−28.8, 27.8)	0.75	3.12 (−23.4, 38.8)	0.84	0.9
N,N-dimethylglycine	−6.65 (−28.5, 21.9)	0.61	1.63 (−22.5, 33.3)	0.91	0.93
Glycine	4.38 (−16.6, 30.7)	0.71	12.9 (−10.2, 41.9)	0.3	0.43
Serine	−2.48 (−21.9, 21.7)	0.82	17.2 (−6.48, 46.9)	0.17	0.29
***Glutathione-related metabolites***					
Total glutathione	−30.1 (−44, −12.8)	0.002	−13.3 (−30.9, 8.88)	0.22	0.36
Total cysteinylglycine	−29.9 (−45.5, −9.9)	0.006	−24.8 (−41.8, −2.85)	0.029	0.067
***Cysteine decarboxylation***					
Taurine	−67.8 (−81, −45.5)	<0.001	−57.3 (−75.1, −27)	0.002	0.008
***Hydrogen sulfide synthesis and catabolism***					
Homolanthionine	−62.3 (−71.9, −49.3)	<0.001	−54.2 (−66.1, −38.1)	<0.001	<0.001
Lanthionine	−25.6 (−40.8, −6.67)	0.011	−2 (−22.3, 23.5)	0.86	0.9
Sulfite	−23.2 (−54.2, 28.9)	0.31	−38.6 (−64.6, 6.56)	0.082	0.17
S-sulfocysteine	−72.8 (−79.3, −64.3)	<0.001	−68.7 (−76.3, −58.7)	<0.001	<0.001
Thiosulfate	−89.4 (−92.8, −84.4)	<0.001	−90.9 (−93.8, −86.4)	<0.001	<0.001
Sulfate	−76.6 (−82.2, −69.3)	<0.001	−75.1 (−81.1, −67.1)	<0.001	<0.001

a Values are derived from linear mixed regression models with log-transformed metabolite as the outcome, and group, visit and their interaction term (group × time) at 4 and 8 weeks as predictors. The models were baseline adjusted. Subject ID was added as a random term to account for within-subject correlation. All estimates are back-transformed and represent % difference vs controls. P-values were derived from the regression models. Adjusted 8-week p-value data were calculated using the Benjamini-Hochberg procedure.

**Table 5:** β-estimates (95 % and confidence intervals) for the excretional fraction of sulfur amino acids and related metabolites in sulfur amino acid restricted individuals vs. controls^a^.

Metabolite	4 weeks		8 weeks		
	β (95 % CI)	p	β (95 % CI)	p	p_adjusted_
***Methionine cycle and transsulfuration***					
Methionine	−11.3 (−25.4, 5.35)	0.17	−17.8 (−30.8, −2.36)	0.026	0.061
Sarcosine (N-methylglycine)	29.1 (−2.46, 70.8)	0.074	46.8 (11.9, 92.5)	0.006	0.019
Cystathionine	−25 (−48.9, 10.1)	0.14	−46.7 (−63.8, −21.5)	0.002	0.006
***Homocysteine remethylation and one-carbon metabolism***					
Choline	−12.1 (−25.2, 3.24)	0.12	−17.4 (−29.6, −2.96)	0.02	0.051
Betaine (N,N,N-trimethylglycine)	−0.225 (−26.6, 35.6)	0.99	−22.7 (−43.1, 4.99)	0.099	0.2
N,N-dimethylglycine	−9.39 (−30.5, 18.1)	0.46	−14.1 (−34.1, 11.9)	0.26	0.4
Glycine	16.6 (−5.51, 44)	0.15	1.84 (−17.5, 25.7)	0.86	0.9
Serine	−3.29 (−17.4, 13.2)	0.67	2.59 (−12.3, 20)	0.75	0.87
***Cysteine decarboxylation***					
Taurine	−71 (−82.8, −51.2)	<0.001	−65.8 (−79.7, −42.5)	<0.001	<0.001
***Hydrogen sulfide synthesis and catabolism***					
Homolanthionine	−30 (−45, −10.8)	0.004	4.05 (−18.3, 32.5)	0.75	0.87
Lanthionine	−10.7 (−26.5, 8.48)	0.25	−1.9 (−19.2, 19.1)	0.85	0.9
S-sulfocysteine	−49.4 (−65.2, −26.5)	<0.001	−35.7 (−55.4, −7.39)	0.018	0.048
Thiosulfate	−79 (−85, −70.6)	<0.001	−73.8 (−81.3, −63.3)	<0.001	<0.001

a Values are derived from linear mixed regression models with log-transformed metabolite as the outcome, and group, visit and their interaction term (group × time) at 4 and 8 weeks as predictors. The models were baseline adjusted. Subject ID was added as a random term to account for within-subject correlation. All estimates are back-transformed and represent % difference vs controls. P-values were derived from the regression models. Adjusted 8-week p-value data were calculated using the Benjamini-Hochberg procedure.

### 3.1 Effects of SAAR on methionine cycle and homocysteine transsulfuration

The Met cycle provides the methyl groups of AdoMet for numerous methylation reactions, and produces homocysteine (Hcy). The effects of SAAR on plasma and urine Met and tHcy have been published previously [29] but are included here for completeness. After 8 weeks on SAAR, we did not observe a significant effect on plasma Met, but a strongly decreased urinary excretion, and indications of a decreased EF (p_adjusted_ = 0.061). There were no conclusive effects of SAAR on plasma AdoMet, AdoHcy or AdoMet/AdoHcy ratio, but tHcy increased in plasma while it significantly decreased in urine. Plasma concentrations of sarcosine were decreased in SAAR vs. controls. The transsulfuration pathway converts Hcy to Cys via the intermediate Csth. The SAAR diet led to a significant decrease of Csth in plasma, urine and its EF. Although tCys was unchanged in plasma, its concentration in urine decreased substantially. These data suggest that SAAR leads to a decreased flow of sulfur metabolites through the Met cycle and the transsulfuration pathway.

### 3.2 Effect of SAAR on Hcy remethylation and one-carbon metabolism

Sulfur metabolism is closely linked to the Hcy remethylation pathways. Chol metabolites, namely Bet, are crucial for Hcy remethylation while Ser and Gly reflect the one-carbon metabolism linked to remethylation. No effects of SAAR were observed on plasma concentrations or 24-h urinary excretion of Chol, Bet, DMG, Gly or Ser. There was a trend for a decreased EF of Chol (p_adjusted_ = 0.051), with no changes in any of the other metabolites. In summary, SAAR had a negligible impact on metabolites in Hcy remethylation and one-carbon metabolism.

### 3.3 Effects of SAAR on glutathione metabolism

GSH is the major intracellular antioxidant and it is synthesized from Cys with γ-glutamylcysteine as an intermediate. A trend for a decrease was observed for the urinary excretion of the catabolic product of GSH degradation – tCysGly (p_adjusted_ = 0.067).

### 3.4 Effects of SAAR on synthesis of Tau from Cys

Cys is an important precursor for synthesis of Tau via the intermediates cysteinesulfinate and HpT. Plasma concentrations of HpT but not Tau decreased in SAAR vs. controls. Urinary output of Tau and its EF decreased significantly in SAAR vs. controls. These data indicate the activation of sulfur-saving strategies by decreasing the decarboxylation pathway of cysteine.

### 3.5 Effects of SAAR on H_2_S synthesis and catabolism

H_2_S is produced from Hcy and Cys via several routes releasing Hlan and Lan as byproducts, respectively, and is further converted to SO_3_^2−^, S_2_O_3_^2−^, and ultimately SO_4_^2−^. Individuals on SAAR showed significantly decreased plasma concentrations of H_2_S compared to controls. Plasma and urinary Hlan were decreased, whereas Lan decreased in plasma only. SO_3_^2−^ was significantly decreased in plasma, with a trend for a decrease also in urine (p_adjusted_ = 0.17). Related metabolites including S_2_O_3_^2−^ and SSC, were also decreased in plasma and urine. Significantly decreased EF was observed for S_2_O_3_^2−^ and SSC. Moreover, urinary excretion of the major end-product of sulfur metabolism, SO_4_^2−^, was significantly decreased to about 25 % in the SAAR group compared to controls. All these data congruently indicate a sulfur saving strategy in the H_2_S synthetic and catabolic pathways in individuals on SAAR.

### 3.6 Estimates of sulfur balance

**Fig. 3:**
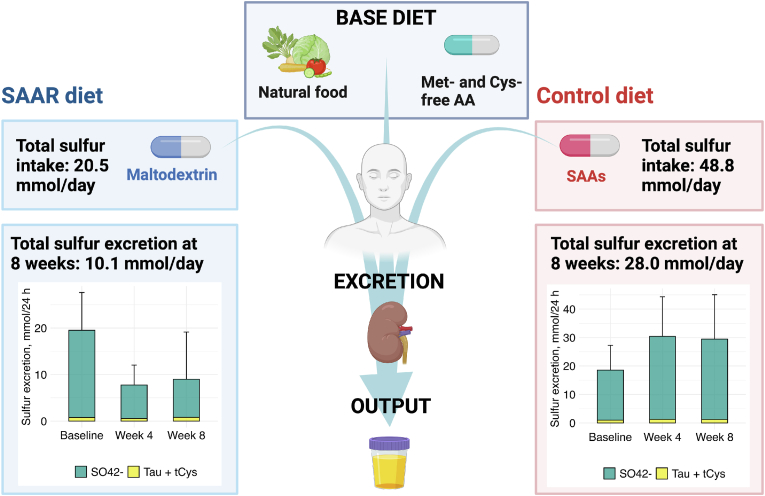
**Sulfur intake and excretion.** The base diet was identical for both groups containing low-protein whole food and capsules with Met-/Cys-free amino acid supplements. The left and right parts show the difference between sulfur intake and output in SAAR and control diet, respectively. Created with BioRender.com.

We estimated the sulfur balance by comparing the sulfur intake in the diet including *ad libitum* food with the excretion of sulfur compounds. A summary figure is shown in Fig. 3. Sulfur intake in the SAAR group were as follows: Met 6.7, cysteine 8.6, Tau 0 and sulfate 5.2 mmol/24-h with a total of 20.5 mmol sulfur/24-h, while the intake in control diet was Met 14.3, Cys 29.3, Tau 0 and sulfate 5.2 with a total of 48.8 mmol sulfur/24-h, respectively. The observed daily excretion of sulfur metabolites was lower than their intake in both diets as shown in Fig. 3. After 8 weeks, the total excretion of sulfur in the SAAR group was −67.6 % (95 % CI: −79.2, −49,5, p_adjusted_ < 0.001). EMMs were (95 % CI) of 10.1 (7.2, 14.2) mmol/24 h in the SAAR group and 28.0 (20.7, 37.8) mmol/24-h in controls. This corresponded to ∼49 % and ∼57 % of the intake in the SAAR group and controls, respectively. After 8 weeks, sulfate was the major form of sulfur excreted representing 92 % and 96 % of all excreted sulfur-containing compounds in the SAAR and control diets, respectively. The sulfur excretion at week 8 between in the SAAR group was 31 % of controls.

### 3.7 Principal component analysis and correlation with phenotypes

**Fig. 4:**
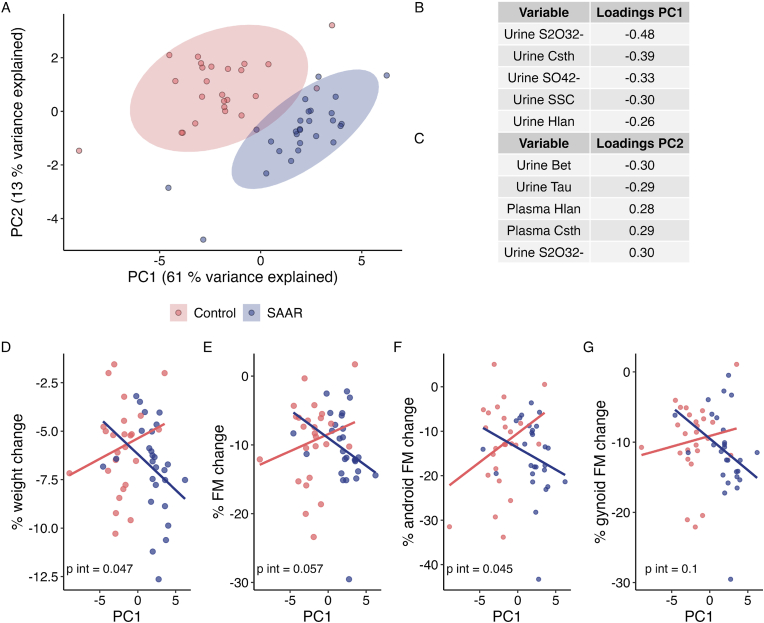
A) Plot of the principal components and corresponding factor loadings (B and C). Panel D through F show the relationship between PC1 and % weight, fat, android fat and gynoid fat change during the intervention. P-values are derived from linear regression models with % change as the outcome and an interaction term for group and PC1 as the predictor.

To gain a more comprehensive understanding about the underlying patterns in the biomarker data, we performed a PCA to identify a sulfurome-related metabolic signature of SAAR. Two PCs were identified that explained 74 % of the variation in the data (Fig. 4A–C). PC1 explained 61 % of the variance. The top 5 factors for PC1 were changes in 24-h urinary excretion of S_2_O_3_^2−^, Csth, SO_4_^2−^, SSC, and Hlan, all of which were negatively correlated to PC1 (Fig. 4B). PC2 explained 13 % of the variance in the dataset. The top 5 associated variables with PC2 were changes in 24-h urinary Bet and Tau (negative), and 24-h urinary S_2_O_3_^2−^ and plasma Csth and Hlan (positive) (Fig. 4C). The SAAR group had significantly higher scores of PC1 and lower scores of PC2 (both p < 0.001). A clear separation between the groups can be seen in Fig. 4A. These exploratory analyses support that sulfur-saving strategies during SAAR involves altered transsulfuration and H_2_S metabolism.

Because we were interested in the underlying mechanism of weight loss during SAAR, we further explored whether the identified PCs were associated with changes in body weight and body fat compartments. Exploratory correlation analyses indicated that PC1 was correlated to changes in body weight and fat mass compartments in the SAAR group only (Appendix Table A.12**).** As such, we assessed whether the effect of SAAR intervention on body weight and body fat compartments depended on the PC1 score (Fig. 4D–G) using linear regression models with interaction terms. There was a significant interaction effect between diet group and PC1 on % weight loss (p for interaction = 0.047) and % android fat mass loss (p for interaction = 0.045), indicating that the difference in weight loss and android fat mass loss between groups increase with increasing PC1 scores. Similar trends were observed for % total fat mass loss (p for interaction = 0.057) and % gynoid fat mass loss (p for interaction = 0.10).

**Fig. 5:**
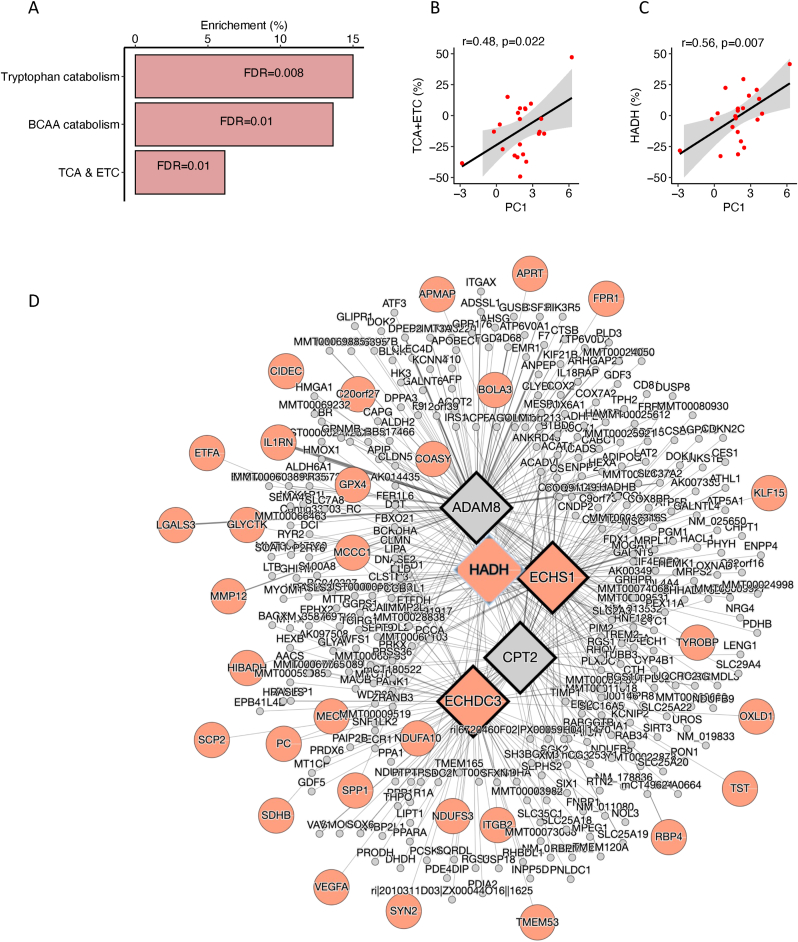
A) Significantly enriched pathways positively associated with PC1 by group interaction term, indicating that the difference between SAAR and controls in expression levels of genes in these pathways increased with greater PC1 scores; B) association between PC1 and change in expression of genes in the tricarboxylic acid pathway and electron transport chain in the SAAR group; C) associations between PC1 and change in *HADH* expression in the SAAR group; D) key driver analysis of the association between PC1 and significantly enriched pathways. Grey, known genes in the network that was not present in the gene set; orange, genes in the network that was present in the gene set; rectangles, key drives of the gene expression changes; circles, the genes associated with the key drivers. (For interpretation of the references to colour in this figure legend, the reader is referred to the Web version of this article.)

Increased scores of PC1 in the SAAR group was correlated with increased enrichment of genes in the TCA cycle and ETC, as well as tryptophan and branched chain amino acid metabolism compared to controls (Fig. 5). The key driver genes of the observed association with the TCA cycle and ETC were *HADH*, *ECHS1* and *ECHDH3* which encodes the enzymes involved mitochondrial β-oxidation. In the SAAR group, PC1 was significantly and positively correlated with the 8-week % change in TCA + ETC mRNA levels from baseline to end-of-study (r = 0.48, p = 0.022) and with % change in *HADH* expression (r = 0.56, p = 0.007).

## 4 Discussion

### 4.1 Key findings

Over an 8-week period, dietary SAAR significantly altered the plasma and urine concentrations of sulfur-containing metabolites in subjects with overweight and obesity. The dietary effect was present at 4 and 8 weeks of the intervention. Metabolites of the transsulfuration pathway, H_2_S production, H_2_S catabolism, and Tau production were substantially decreased in both plasma and urine of the SAAR group compared to the control group, with the clearest effects observed in 24-h urinary output. This decrease is evident in the approximately 69 % reduction in total sulfur excretion at the end of intervention in the SAAR group compared to controls. By using PCA, we identified a distinct metabolic signature associated with SAAR, characterized by a notable decrease in the urinary excretion of intermediates involved in transsulfuration and H_2_S metabolism. Exploratory analyses showed that this signature correlated negatively with change in body weight (i.e., higher scores, greater body weight loss) and body fat mass compartments.

### 4.2 Cys catabolism and sulfur excretion

There are two major pathways of cysteine catabolism (reviewed in Refs. [[40], [41], [42]]). The first pathway involves oxidation to cysteinesulfinate, followed by subsequent conversion to HpT and Tau. Cysteine dioxygenase 1 (CDO1) catalyzes the rate-limiting step in this pathway, involving the oxidation of Cys to cysteinesulfinate, which can also produce SO_3_^2−^ and the end-product SO_4_^2^. CDO1 exhibits a high specificity for cysteine [43] and is highly responsive to changes in SAA intake, as demonstrated in animal experiments (reviewed in Ref. [41]). Alternatively, Cys can undergo desulfhydration to produce H_2_S, which is then further oxidized to SO_4_^2−^ (Fig. 1). Animal studies have extensively described these pathways [41], and data from a human study suggest that populations with low Cys intake, such as vegans, exhibit lower urinary excretion of Tau [44]. Here, we focused solely on the chronic dietary manipulation of the SAA and our data strongly suggests the involvement of these catabolic pathways in humans. Dietary SAAR led to significant reductions in plasma concentrations of HpT, H_2_S, S_2_O_3_^2−^, and SO_3_^2−^. Moreover, there was a marked decrease in urinary excretion of Tau, S_2_O_3_^2−^ and SO_4_^2−^. Collectively, our results indicate that the Cys catabolic machinery is inhibited to preserve Cys availability. Furthermore, the decreased urinary excretion of tCys in the SAAR group, along with the observed findings, may explain the consistent lack of effects of SAAR on plasma tCys in our previous pilot studies [45,46]. Another potential mechanism that may contribute to the maintenance of plasma tCys during SAAR is autophagy [47]. Autophagy leads to proteolysis that may in turn liberate the Cys-Cys disulfide cystine [48]. Interestingly, we have previously observed a trend for an increase in plasma cystine (p = 0.06) during short-term SAAR in normal-weight individuals [45]. However, it should be noted that cystine constitutes around only 15 % of plasma tCys, and further studies are therefore required. Unfortunately, we were not able to quantify plasma cystine in the present study.

Several other observations in the present study also warrant discussion. We observed a paradoxical increase in plasma total tHcy concentrations in the SAAR group compared to controls. This observation has been previously reported in animal and human studies on SAAR [45,46,49,50], and is likely due to reduced availability of Met and thereby AdoMet, which inhibits cystathionine β-synthase (CBS) activation and degradation of Hcy. Indeed, Csth, the product of the CBS reaction is substantially decreased in plasma and urine when dietary Met is limited [29,45,46]. Although plasma AdoMet was unchanged compared to controls, we observed a considerable decrease in sarcosine. Sarcosine is produced by glycine N-methyltransferase (GNMT) in situations of AdoMet excess, and decreased sarcosine thus indicate that AdoMet levels are being maintained in plasma during SAAR by downregulation of GNMT. We also demonstrated a decrease in plasma and urine levels of Hlan, a product of H_2_S production from Hcy, further indicating that Hcy is conserved for Met synthesis when dietary SAA are low.

Despite the expected decrease in total sulfur excretion in SAAR vs. controls, SO_4_^2−^ remains the dominant excretion product of dietary sulfur intake (>90 % in both groups). Of the total sulfur excretion, there was a slight increase in Tau and tCys in the control group, likely reflecting increased Cys catabolism and renal handling in situations where SAA are consumed in excess. The contribution of other sulfur compounds to total sulfur excretion was negligible. The difference between SAA intake in the SAAR and control diet was 28.2 mmol/24-h, however, the difference of only 20.1 mmol of SO_4_^2−^ excretion/24-h and only 0.6 mmol/24-h of other sulfur-containing metabolites indicate that SAA are still used for anabolism in both diets. These data collectively indicate that catabolism of SAA to SO_4_^2−^ via CDO1 represent the major mechanism of regulating SAA metabolism in humans.

### 4.3 The sulfurome, weight loss and adipose tissue gene expression

We have previously reported that dietary SAAR induce weight reduction in humans [29], which aligns with findings from animal studies [[51], [52], [53], [54]]. Recent studies have highlighted the direct involvement of sulfur metabolism in mediating such beneficial effects, implicating both increased production capacity and intracellular concentrations of H_2_S and the direct effects of Cys on PPARγ -mediated adipogenesis [[22], [23], [24], [25],^55^]. This increased H_2_S production capacity of cells observed during SAAR may seem paradoxical given the limited availability of its main precursor, Cys. However, recent findings indicate that cystathionine γ-lyase (CTH) can utilize the cysteine-cysteine disulfide cystine liberated during SAAR-induced autophagy as a substrate for H_2_S production [56]. In contrast to animal studies, our results do not support an increase in H_2_S production during SAAR. In fact, plasma H_2_S concentrations were reduced by 25 % in the SAAR group compared to controls. Additionally, by-products of H_2_S synthesis, Hlan and Lan, were similarly decreased in plasma and urine, whereas products of H_2_S catabolism showed a reduction of up to 75 %. Collectively, these findings indicate a decrease rather than an increase in H_2_S production during SAAR in humans. There are several potential reasons for this discrepancy with the animal literature. In animal studies, experimental diets completely lack Cys, which is not possible to achieve in human interventions. In addition, the reported plasma H_2_S concentrations vary considerably in different studies illustrating major differences in the analytical methods that are applied for H_2_S determination [[57], [58], [59]]. It is important to note that we here report bioavailable H_2_S concentrations as measured with our improved monobromobimane method [57].

In exploratory PCA analyses, we identified a metabolic signature of SAAR explaining >60 % of the variation in the dataset (PC1), that correlated with weight loss and loss of fat mass in the SAAR group. PC1 was largely explained by decreased urinary excretion of metabolites indicative of decreased transsulfuration and decreased desulfhydration. Interaction analyses suggested that the effect of SAAR on weight and fat mass loss was greater with higher scores of PC1, i.e., presumably lower transsulfuration and Hcy/Cys desulfhydration as a result of greater degree of dietary restriction. It remains an open question whether this signature reflects different genetic background, or whether these metabolic pathways take active part in weight loss effects of SAAR in humans. Interestingly, supplementation of H_2_S donors in *in vitro* studies enhances adipogenesis, whereas inhibition of H_2_S production capacity severely suppresses this process and decreases lipogenesis and fat storage [24,60]. Such results suggest that decreased Cys availability can limit lipogenesis by suppressing H_2_S production capacity, possibly by interfering with the expression of PPARγ target genes [24]. Human studies are scarce, but a recent study reported that plasma sulfide concentrations were increased in obese compared to non-obese individuals and that plasma concentrations decreased with weight loss [61]. The authors also reported a trend for higher mRNA expression of the H_2_S-producing enzyme CBS in whole blood of individuals with obesity (p = 0.09). As such, there are indications from the literature that H_2_S metabolism may be involved in the process of obesity development, which coincides with our reported correlation of seemingly decreased transsulfuration and desulfhydration with increased weight and fat mass loss. However, it should be noted that H_2_S is also inversely associated with type 2 diabetes [62], and has been considered beneficial in obesity development [63], reflecting the uncertainty of this concept. In order to better understand these processes, experimental studies could focus on describing the effect of SAAR on tissue metabolism of H_2_S, particularly in diet-induced obesity models which may hold more relevance to humans where studies take place in individuals with overweight and obesity.

Whether the effects of SAAR on body weight or WAT gene expression are directly linked to sulfurome changes in WAT remains to be investigated, since the WAT sulfurome could not be assayed in the present study. Despite the known elevation of plasma tCys [11,64], and its non-protein bound disulfide form, cystine in plasma and blood from people with obesity [9,65,66], a contrasting finding was observed in one study. Specifically, the concentrations of cystine and methionine were found to be 40 % lower in WAT from obese vs. lean individuals [67]. This suggests that the concentrations of sulfur metabolites in adipose tissue may not necessarily track plasma levels, and may not be the key driver of the WAT changes mediating the body weight phenotypes. Notably, we did not observe any associations for expression of WAT *CBS* and *CTH* with plasma levels of tHcy or tCys (data not shown).

We leveraged transcriptomic data to further investigate whether the changes observed in the sulfurome correlated with gene expression in scWAT. We have previously reported that SAAR on average led to small, but significant decreases in the expression of genes related to lipid storage compare to controls [29]. Here, we report that increasing scores of the PC1, a signature indicative of reduced flux through these pathways, correlated with processes related to energy metabolism in scWAT including the TCA cycle and ETC. These processes were driven by enzymes related to mitochondrial β-oxidation of fatty acids. Our findings are in line with animal studies demonstrating increased WAT expression of genes in β-oxidation, and several-fold increased induction of fatty acid oxidation and TCA cycle activity WAT in response to SAAR [68]. This effect is thought to contribute to the transcriptional remodeling of adipose tissue observed in such experiments. Unfortunately, we were not able to quantify adipocyte size in the current study.

### 4.4 Strengths and limitations

The main strength of this study is the randomized, double-blind design which is rare in dietary intervention studies. Another strength is the rigorous and sensitive analytical approach applied to measure several sulfur metabolites in plasma and urine in the nanomolar range. Excretion of analytes in 24-h urine appears to reflect the dietary intervention to a larger extent than the less apparent changes in plasma. This study also has limitations. Considering that the study was conducted in individuals with overweight or obesity with a majority of women, generalizability is limited. An additional limitation is that *a priori* power calculations for the outcomes reported herein were not performed. Although the analytical methods performed generally well, determination of SO_3_^2−^ was sensitive to interferences, which prevented evaluation of sulfite in 20 % and 59 % plasma and urinary samples, respectively. Finally, although we have comprehensively analyzed intermediates of SAA metabolism, lack of tissue and/or tracer data limit any inference on metabolic processes.

### 4.5 Conclusion

In conclusion, dietary SAAR have marked effects on plasma and urine concentrations, and excretional fraction of sulfur-containing metabolites in individuals with overweight or obesity. The effects were particularly pronounced on urinary concentrations markers of transsulfuration, taurine synthesis and desulfhydration/H_2_S metabolism, which was also reflected in a metabolic signature as identified by PCA. It is unclear whether changes in sulfur metabolism is contributing to the weight loss, and future experimental and clinical studies are needed to address these topics.

## Funding

Open access funding provided by University of Oslo (incl Oslo University Hospital). This project has received funding from The Research Council of Norway (Grant no: 310475) under the umbrella of the European Joint Programming Initiative “A Healthy Diet for a Healthy Life” (JPI HDHL) and of the ERA-NET Cofund HDHL INTIMIC (GA N° 727565 of the EU Horizon 2020 Research and Innovation Programme), Institute of Basic Medical Sciences, University of Oslo, and Henning och Johan Throne-Holsts stiftelse. VK and KB were supported by the Czech Ministry of Education, Sports and Youth (STAY 8F20013), institutional support was provided by Charles University (program COOPERATIO-Metabolic Disorders) and Ministry of Health DRO VFN64165.

## CRediT authorship contribution statement

**Thomas Olsen:** Writing – review & editing, Writing – original draft, Visualization, Supervision, Project administration, Methodology, Investigation, Funding acquisition, Formal analysis, Data curation, Conceptualization. **Kathrine J. Vinknes:** Writing – review & editing, Writing – original draft, Supervision, Project administration, Methodology, Investigation, Funding acquisition, Data curation, Conceptualization. **Kristýna Barvíková:** Writing – review & editing, Writing – original draft, Visualization, Methodology, Formal analysis, Data curation. **Emma Stolt:** Writing – review & editing, Project administration, Investigation, Data curation. **Sindre Lee-Ødegård:** Writing – review & editing, Visualization, Formal analysis, Data curation. **Hannibal Troensegaard:** Writing – review & editing, Investigation, Data curation. **Hanna Johannessen:** Writing – review & editing, Investigation, Data curation. **Amany Elshorbagy:** Writing – review & editing, Methodology, Funding acquisition, Conceptualization. **Jitka Sokolová:** Writing – review & editing, Methodology, Investigation. **Jakub Krijt:** Writing – review & editing, Methodology, Investigation. **Michaela Křížková:** Writing – review & editing, Methodology, Investigation. **Tamás Ditrói:** Writing – review & editing, Methodology, Data curation. **Péter Nagy:** Writing – review & editing, Methodology, Data curation. **Bente Øvrebø:** Writing – review & editing, Methodology, Conceptualization. **Helga Refsum:** Writing – review & editing, Supervision, Methodology, Conceptualization. **Magne Thoresen:** Writing – review & editing, Formal analysis, Conceptualization. **Kjetil Retterstøl:** Writing – review & editing, Supervision, Methodology, Funding acquisition, Conceptualization. **Viktor Kožich:** Writing – review & editing, Writing – original draft, Supervision, Methodology, Investigation, Funding acquisition, Conceptualization.

## Declaration of competing interest

The authors declare that they have no known competing financial interests or personal relationships that could have appeared to influence the work reported in this paper.

## Data Availability

Due to European privacy legislations, data in this article cannot be made publicly available. Data will be shared upon request, pendig institutional collaboration agreements and upon presentation of relevant ethical approvals and approvals from data protection authorities.
