# Supplementary material for: Dietary sulfur amino acid restriction in humans with overweight and obesity: Evidence of an altered plasma and urine sulfurome, and a novel metabolic signature that correlates with loss of fat mass and adipose tissue gene expression
Source: Redox Biol. 2024 May 17;73:103192. doi: 10.1016/j.redox.2024.103192 (PMC11163171; doi:10.1016/j.redox.2024.103192)
Supplement: Multimedia component 1 [file mmc1.docx]

**Dietary sulfur amino acid restriction in humans with overweight and obesity: Evidence of an altered plasma and urine sulfurome, and a novel metabolic signature that correlates with loss of fat mass and adipose tissue gene expression**

Thomas Olsen^1,*^, Kathrine J. Vinknes^1,#^, Kristýna Barvíková^2,#^, Emma Stolt^1^, Sindre Lee-Ødegård^3^, Hannibal Troensegaard^1^, Hanna Johannessen^4^, Amany Elshorbagy^5,6^, Jitka Sokolová^2^, Jakub Krijt^2^, Michaela Křížková^2^, Tamás Ditrói^7^, Péter Nagy^7,8,9^, Bente Øvrebø^1,10^, Helga Refsum^1,6^, Magne Thoresen^11^, Kjetil Retterstøl^1,12^, Viktor Kožich^2,*^

^1^ Department of Nutrition, Institute of Basic Medical Sciences, Faculty of Medicine University of Oslo, Postboks 1046 Blindern, 0317 Oslo, Norway

^2^ Department of Pediatrics and Inherited Metabolic Disorders, Charles University, First Faculty of Medicine, and General University Hospital, Ke Karlovu 2, 128 00 Prague, Czech Republic

^3^ Department of Endocrinology, Morbid Obesity and Preventive Medicine, Institute of Clinical Medicine, Faculty of Medicine, University of Oslo, Postboks 4959 Nydalen, OUS HF Aker sykehus, 0424 Oslo, Norway

^4^ Department of Pathology, Oslo University Hospital, Rikshospitalet, Postboks 45980 Nydalen, OUS HF Rikshospitalet, 0424 Oslo, Norway

^5^ Department of Physiology, Faculty of Medicine, University of Alexandria, Chamblion street, Qesm Al Attarin, Alexandria 5372066, Egypt

^6^ Department of Pharmacology, University of Oxford, Mansfield Rd, Oxford OX1 3QT, UK

^7^ Department of Molecular Immunology and Toxicology and the National Tumor Biology Laboratory, National Institute of Oncology, Ráth György u. 7-9, 1122 Budapest, Hungary

^8^ Department of Anatomy and Histology, HUN-REN–UVMB Laboratory of Redox Biology Research Group, University of Veterinary Medicine, 1078 Budapest, Hungary

^9^ Chemistry Institute, University of Debrecen, 4012 Debrecen, Hungary

^10^ Department of Food Safety, Norwegian Institute of Public Health, Postboks 222 Skøyen, 0213 Oslo, Norway

^11^ Department of Biostatistics, Institute of Basic Medical Sciences, University of Oslo, Postboks 1122 Blindern, 0317 Oslo, Norway

^12^ The Lipid Clinic, Department of Endocrinology, Morbid Obesity and Preventive Medicine, Oslo University Hospital, Postboks 4959 Nydalen, OUS HF Aker sykehus, 0424 Oslo, Norway

* Correspondence: Thomas Olsen, [thomas.olsen@medisin.uio.no](mailto:thomas.olsen@medisin.uio.no) or Viktor Kožich, [viktor.kozich@vfn.cz](mailto:viktor.kozich@vfn.cz)

^#^ Shared authorship

**Supplemental methods**

*Blood sampling and biochemical analyses of histidine and pyruvate*

For histidine and pyruvate blood was collected into a 4 mL EDTA tube. These were centrifuged for 10 min at 2500 *g* at 4 °C. Samples for amino acid, pyruvate, histidine and alanine assays were stored at – 80 °C for up to 3 months prior to analysis. Histidine, alanine and pyruvate were determined by NMR.

| **Appendix Table A.1:** Baseline characteristics of the participants in the SAAR and control groups^1^ | | |
| --- | --- | --- |
| **Characteristics** | **SAAR**  (n = 31) | **Controls**  (n = 28) |
| Males, n (%) | 8 (25.8) | 8 (28.6) |
| Age, y | 33.2 (5.86) | 34.4 (6.26) |
| Body weight, kg | 94.4 (10.7) | 88.7 (9.22) |
| Body mass index, kg/m^2^ | 31.4 (2.12) | 30.8 (2.20) |
| Waist-to-hip ratio | 0.85 (0.08) | 0.86 (0.08) |
| Percent body fat, % | 42.0 (6.76) | 42.4 (5.80) |
| Systolic blood pressure, mm/Hg | 120 (10.9) | 119 (10.7) |
| Diastolic blood pressure, mm/Hg | 69.1 (7.57) | 70.0 (8.50) |
| Glucose, mmol/L | 5.16 (0.41) | 5.31 (0.50) |
| Insulin, pmol/L | 81.0 (47.0) | 75.5 (39.9) |
| ^1^Abbrevations: SAAR, sulfur amino acid restriction; All variables are mean (standard deviation). | | |

| **Appendix Table A.2:** Unadjusted geometric mean (geometric standard deviation) concentrations of sulfur amino acids and related metabolites in plasma (μmol/L). | | | | |
| --- | --- | --- | --- | --- |
|  |  | Baseline | 4 weeks | 8 weeks |
| Methionine | SAAR | 21 (1.18) | 20.4 (1.24) | 20.1 (1.21) |
|  | Controls | 22.4 (1.19) | 21.7 (1.25) | 22.0 (1.16) |
| S-adenosylmethionine | SAAR | 0.0651 (1.19) | 0.0667 (1.15) | 0.0661 (1.15) |
|  | Controls | 0.0649 (1.18) | 0.0671 (1.15) | 0.0673 (1.15) |
| S-adenosylhomocysteine | SAAR | 0.0206 (1.45) | 0.0176 (1.45) | 0.0177 (1.31) |
|  | Controls | 0.0199 (1.3) | 0.017 (1.26) | 0.017 (1.42) |
| Total homocysteine | SAAR | 9.29 (1.32) | 9.61 (1.34) | 9.81 (1.36) |
|  | Controls | 9.43 (1.3) | 8.12 (1.31) | 8.23 (1.25) |
| Cystathionine | SAAR | 0.226 (1.71) | 0.117 (1.47) | 0.124 (1.67) |
|  | Controls | 0.206 (1.49) | 0.228 (1.87) | 0.244 (1.75) |
| Total cysteine | SAAR | 261 (1.14) | 266 (1.1) | 268 (1.12) |
|  | Controls | 272 (1.16) | 261 (1.1) | 265 (1.12) |
| Total glutathione | SAAR | 6.35 (1.28) | 6.43 (1.27) | 6.60 (1.3) |
|  | Controls | 6.46 (1.28) | 6.49 (1.27) | 6.21 (1.26) |
| Total cysteinylglycine | SAAR | 25.1 (1.26) | 24.8 (1.21) | 24.8 (1.23) |
|  | Controls | 24.9 (1.25) | 23.2 (1.2) | 23.6 (1.24) |
| Total γ-glutamylcysteine | SAAR | 3.83 (1.22) | 4.01 (1.15) | 3.96 (1.2) |
|  | Controls | 4.05 (1.25) | 4.04 (1.17) | 3.99 (1.2) |
| Cysteamine | SAAR | 0.0193 (1.54) | 0.0196 (1.15) | 0.0219 (1.31) |
|  | Controls | 0.0218 (1.15) | 0.0176 (1.18) | 0.0183 (1.29) |
| Hypotaurine | SAAR | 0.755 (1.4) | 0.631 (1.45) | 0.653 (1.53) |
|  | Controls | 0.709 (1.39) | 0.968 (1.34) | 0.995 (1.45) |
| Taurine | SAAR | 56.3 (1.24) | 51.7 (1.3) | 52.8 (1.32) |
|  | Controls | 55.8 (1.29) | 52.6 (1.22) | 55.4 (1.25) |
| S-sulfocysteine | SAAR | 0.138 (1.25) | 0.126 (1.29) | 0.139 (1.28) |
|  | Controls | 0.149 (1.22) | 0.255 (1.43) | 0.302 (1.6) |
| Pyruvate | SAAR | 29.4 (1.51) | 27.8 (1.5) | 25.2 (1.46) |
|  | Controls | 29.8 (1.44) | 24 (1.4) | 28.3 (1.66) |
| Sulfide, bioavailable | SAAR | 0.0517 (1.29) | 0.0468 (1.38) | 0.039 (1.76) |
|  | Controls | 0.0475 (1.3) | 0.0532 (1.4) | 0.0488 (1.45) |
| Lanthionine | SAAR | 0.0518 (1.43) | 0.0476 (1.41) | 0.0517 (1.37) |
|  | Controls | 0.0548 (1.23) | 0.0585 (1.32) | 0.0623 (1.32) |
| Homolanthionine | SAAR | 0.00896 (1.67) | 0.00551 (1.56) | 0.00521 (1.75) |
|  | Controls | 0.00847 (1.45) | 0.0109 (1.91) | 0.0121 (1.9) |
| Thiosulfate | SAAR | 0.299 (1.3) | 0.258 (1.21) | 0.265 (1.21) |
|  | Controls | 0.312 (1.22) | 0.475 (1.54) | 0.516 (1.62) |
| Sulfite | SAAR | 0.0525 (1.97) | 0.0499 (1.99) | 0.049 (2.00) |
|  | Controls | 0.052 (2.17) | 0.0725 (1.92) | 0.0822 (2.06) |
| Choline | SAAR | 6.03 (1.27) | 5.14 (1.23) | 5.39 (1.25) |
|  | Controls | 6.24 (1.25) | 5.63 (1.22) | 5.91 (1.22) |
| Betaine | SAAR | 25.7 (1.62) | 27.8 (1.7) | 30.7 (1.69) |
|  | Controls | 27.1 (1.44) | 28.8 (1.41) | 30.9 (1.3) |
| Dimethylglycine | SAAR | 3.47 (1.38) | 3.43 (1.36) | 3.75 (1.32) |
|  | Controls | 3.51 (1.39) | 3.47 (1.33) | 3.65 (1.29) |
| Sarcosine | SAAR | 1.13 (1.44) | 0.823 (1.54) | 0.823 (1.46) |
|  | Controls | 1.22 (1.42) | 1.22 (1.29) | 1.34 (1.31) |
| Glycine | SAAR | 207 (1.36) | 232 (1.4) | 236 (1.41) |
|  | Controls | 224 (1.35) | 251 (1.34) | 257 (1.36) |
| Serine | SAAR | 98.2 (1.19) | 114 (1.22) | 110 (1.21) |
|  | Controls | 101 (1.24) | 112 (1.22) | 109 (1.22) |
| Alanine | SAAR | 295 (1.21) | 286 (1.24) | 273 (1.21) |
|  | Controls | 327 (1.21) | 307 (1.21) | 302 (1.20) |
| Glutamate | SAAR | 28.3 (1.68) | 27.5 (1.66) | 22.7 (1.52) |
|  | Controls | 31.3 (1.88) | 29.4 (1.77) | 27.9 (1.86) |
| Glutamine | SAAR | 380 (1.16) | 424 (1.22) | 411 (1.19) |
|  | Controls | 402 (1.23) | 416 (1.24) | 410 (1.2) |
| Histidine | SAAR | 69.6 (1.13) | 70.6 (1.07) | 71.3 (1.12) |
|  | Controls | 72.2 (1.14) | 72.3 (1.13) | 68.4 (1.17) |
| Abbreviations: SAAR, sulfur amino acid restriction | | | | |

| **Appendix Table A.3:** Unadjusted geometric mean (geometric standard deviation) of urine sulfur amino acids and related metabolites in 24-h urine (μmol/24 h) | | | | |
| --- | --- | --- | --- | --- |
|  |  | Baseline | 4 weeks | 8 weeks |
| Methionine | SAAR | 8.92 (1.86) | 5.29 (1.71) | 5.67 (1.87) |
|  | Controls | 7.42 (1.98) | 7.99 (1.65) | 7.76 (1.68) |
| Total homocysteine | SAAR | 8.27 (1.67) | 5.57 (1.56) | 5.16 (1.59) |
|  | Controls | 6.77 (1.83) | 7.59 (1.58) | 6.88 (1.69) |
| Cystathionine | SAAR | 36.6 (2.16) | 6.34 (2.05) | 6.61 (2.39) |
|  | Controls | 26.5 (2.21) | 24 (2.35) | 27.7 (2.28) |
| Total cysteine | SAAR | 243 (1.5) | 227 (1.53) | 202 (1.44) |
|  | Controls | 216 (1.54) | 277 (1.38) | 254 (1.56) |
| Total cysteinylglycine | SAAR | 8.42 (1.58) | 5.88 (1.79) | 5.78 (1.63) |
|  | Controls | 7.61 (1.57) | 7.94 (1.66) | 7.22 (1.65) |
| Cysteamine | SAAR | 0.555 (1.68) | 0.603 (1.76) | 0.512 (2.94) |
|  | Controls | 0.529 (1.5) | 0.659 (1.46) | 0.661 (1.46) |
| Taurine | SAAR | 316 (3.53) | 180 (3.23) | 250 (4.06) |
|  | Controls | 399 (3.48) | 596 (2.8) | 622 (2.59) |
| S-sulfocysteine | SAAR | 6.53 (1.56) | 4.43 (1.48) | 4.65 (1.82) |
|  | Controls | 5.72 (1.57) | 15.3 (1.73) | 13.6 (2.16) |
| Lanthionine | SAAR | 14.3 (1.77) | 12.4 (1.51) | 13.1 (1.65) |
|  | Controls | 13.2 (1.62) | 15.9 (1.42) | 13.3 (1.58) |
| Homolanthionine | SAAR | 1.33 (1.7) | 0.84 (1.84) | 0.921 (1.86) |
|  | Controls | 1.07 (1.7) | 2.05 (1.77) | 1.89 (1.84) |
| Thiosulfate | SAAR | 16.6 (1.71) | 7.44 (1.68) | 7.35 (1.89) |
|  | Controls | 13.9 (1.99) | 65.9 (2.82) | 71.4 (2.8) |
| Sulfate | SAAR | 17100 (1.62) | 6380 (1.65) | 6390 (1.86) |
|  | Controls | 15700 (1.68) | 26600 (1.62) | 24600 (1.7) |
| Sulfite | SAAR | 1.79 (2.7) | 1.88 (3.23) | 2.54 (2.94) |
|  | Controls | 1.87 (2.98) | 3.03 (2.24) | 3.90 (2.16) |
| Choline | SAAR | 24.5 (1.76) | 17.9 (1.41) | 18.1 (1.52) |
|  | Controls | 23.3 (1.57) | 20.7 (1.48) | 19.5 (1.48) |
| Betaine | SAAR | 60.2 (2.12) | 36.8 (1.85) | 41.4 (2.23) |
|  | Controls | 55.2 (1.91) | 34.9 (1.68) | 39.6 (1.76) |
| Dimethylglycine | SAAR | 60.2 (2.26) | 39.4 (1.84) | 41.9 (2.13) |
|  | Controls | 53.8 (1.99) | 37.4 (1.98) | 40.1 (1.98) |
| Sarcosine | SAAR | 1.82 (1.97) | 1.39 (1.61) | 1.53 (1.70) |
|  | Controls | 1.96 (1.97) | 1.35 (1.69) | 1.40 (1.83) |
| Glycine | SAAR | 1660 (1.84) | 1470 (1.65) | 1510 (1.69) |
|  | Controls | 1680 (1.68) | 1330 (1.53) | 1370 (1.54) |
| Serine | SAAR | 405 (1.88) | 378 (1.6) | 385 (1.73) |
|  | Controls | 356 (1.65) | 337 (1.45) | 294 (1.62) |
| Alanine | SAAR | 307 (1.83) | 187 (1.67) | 194 (1.89) |
|  | Controls | 281 (1.76) | 165 (1.5) | 158 (1.48) |
| Glutamate | SAAR | 28.2 (1.53) | 28.7 (1.59) | 28.6 (1.88) |
|  | Controls | 28.7 (1.53) | 22.9 (1.44) | 21.5 (1.59) |
| Glutamine | SAAR | 505 (1.77) | 400 (1.56) | 402 (1.76) |
|  | Controls | 430 (1.67) | 314 (1.51) | 285 (1.49) |
| Abbreviations: SAAR, sulfur amino acid restriction | | | | |

| **Appendix Table A.4:** Unadjusted geometric mean (geometric standard deviation) of sulfur amino acids and related metabolites in excretional fraction (%) | | | | |
| --- | --- | --- | --- | --- |
|  |  | Baseline | 4 weeks | 8 weeks |
| Methionine | SAAR | 0.243 (1.4) | 0.179 (1.35) | 0.196 (1.34) |
|  | Controls | 0.235 (1.45) | 0.197 (1.35) | 0.229 (1.48) |
| Cystathionine | SAAR | 76.9 (1.83) | 30.5 (2.07) | 27.3 (1.83) |
|  | Controls | 74.4 (1.85) | 40.1 (2.37) | 52.1 (2.58) |
| Cysteamine | SAAR | 11.7 (1.41) | 13.9 (1.67) | 14.1 (1.25) |
|  | Controls | 14.4 (1.13) | 23.2 (1.31) | 22.8 (1.3) |
| Taurine | SAAR | 2 (3.64) | 1.03 (3.55) | 1.39 (3.37) |
|  | Controls | 2.83 (2.93) | 4.07 (3.25) | 4.12 (2.97) |
| S-sulfocysteine | SAAR | 18.3 (1.49) | 13.9 (1.67) | 13.4 (1.6) |
|  | Controls | 17.4 (1.37) | 27 (2.1) | 19.9 (1.8) |
| Lanthionine | SAAR | 132 (1.52) | 138 (1.59) | 136 (1.44) |
|  | Controls | 125 (1.39) | 150 (1.55) | 138 (1.67) |
| Homolanthionine | SAAR | 80 (1.56) | 78.6 (1.51) | 93.5 (1.63) |
|  | Controls | 72.3 (1.42) | 110 (1.66) | 89.7 (1.66) |
| Thiosulfate | SAAR | 25.7 (1.68) | 13.8 (1.63) | 13.5 (1.57) |
|  | Controls | 24.2 (1.45) | 65.6 (2.52) | 48.9 (2.43) |
| Choline | SAAR | 1.76 (1.42) | 1.51 (1.43) | 1.51 (1.39) |
|  | Controls | 1.83 (1.33) | 1.74 (1.36) | 1.81 (1.38) |
| Betaine | SAAR | 0.938 (2.08) | 0.523 (2.19) | 0.549 (2.75) |
|  | Controls | 0.931 (1.73) | 0.523 (1.64) | 0.709 (1.53) |
| Dimethylglycine | SAAR | 7.87 (2.05) | 4.91 (2) | 5.11 (2.44) |
|  | Controls | 6.75 (1.8) | 4.72 (2.1) | 5.54 (2.03) |
| Sarcosine | SAAR | 0.743 (1.87) | 0.607 (1.96) | 0.699 (1.7) |
|  | Controls | 0.684 (2.03) | 0.449 (1.56) | 0.446 (1.81) |
| Glycine | SAAR | 3.4 (1.72) | 2.73 (1.51) | 2.74 (1.53) |
|  | Controls | 3.46 (1.81) | 2.17 (1.55) | 2.59 (1.5) |
| Serine | SAAR | 2.04 (1.58) | 1.69 (1.53) | 1.89 (1.36) |
|  | Controls | 1.87 (1.55) | 1.55 (1.26) | 1.62 (1.26) |
| Alanine | SAAR | 0.456 (1.64) | 0.274 (1.7) | 0.324 (1.78) |
|  | Controls | 0.395 (1.71) | 0.246 (1.45) | 0.281 (1.49) |
| Glutamate | SAAR | 0.469 (1.8) | 0.477 (1.87) | 0.621 (1.89) |
|  | Controls | 0.427 (2.05) | 0.355 (1.94) | 0.45 (2.01) |
| Glutamine | SAAR | 0.624 (1.54) | 0.484 (1.53) | 0.517 (1.46) |
|  | Controls | 0.533 (1.62) | 0.375 (1.3) | 0.436 (1.3) |
| Abbreviations: SAAR, sulfur amino acid restriction | | | | |

| **Appendix Table A.5.** Estimated marginal means of plasma sulfur amino acids (μmol/L) and related metabolites^1^ | | | | |
| --- | --- | --- | --- | --- |
|  |  | Baseline | 4 weeks | 8 weeks |
| Methionine | SAAR | 22.2 (21.1, 23.4) | 21.3 (20, 22.7) | 21 (19.7, 22.4) |
|  | Control | 22.2 (21.1, 23.4) | 21.9 (20.5, 23.5) | 22.3 (20.9, 23.8) |
| S-adenosylmethionine | SAAR | 0.065 (0.062, 0.068) | 0.067 (0.063, 0.07) | 0.066 (0.063, 0.07) |
|  | Control | 0.065 (0.062, 0.068) | 0.067 (0.063, 0.071) | 0.067 (0.063, 0.07) |
| Sarcosine (N-methylglycine) | SAAR | 1.19 (1.08, 1.31) | 0.86 (0.76, 0.98) | 0.87 (0.77, 0.99) |
|  | Control | 1.19 (1.08, 1.31) | 1.23 (1.08, 1.4) | 1.33 (1.18, 1.51) |
| S-adenosylhomocysteine | SAAR | 0.021 (0.019, 0.023) | 0.018 (0.016, 0.02) | 0.018 (0.016, 0.02) |
|  | Control | 0.021 (0.019, 0.023) | 0.017 (0.015, 0.02) | 0.018 (0.016, 0.02) |
| Total homocysteine | SAAR | 9.77 (9.06, 10.5) | 10.2 (9.33, 11.1) | 10.5 (9.65, 11.5) |
|  | Control | 9.77 (9.06, 10.5) | 8.3 (7.6, 9.06) | 8.5 (7.8, 9.26) |
| Cystathionine | SAAR | 0.22 (0.20, 0.26) | 0.12 (0.10, 0.15) | 0.13 (0.10, 0.15) |
|  | Control | 0.22 (0.20, 0.26) | 0.24 (0.20, 0.29) | 0.26 (0.22, 0.32) |
| Total cysteine | SAAR | 269 (261, 279) | 273 (262, 285) | 278 (266, 290) |
|  | Control | 269 (261, 279) | 260 (248, 271) | 265 (254, 277) |
| Choline | SAAR | 6.14 (5.78, 6.53) | 5.19 (4.79, 5.63) | 5.46 (5.03, 5.93) |
|  | Control | 6.14 (5.78, 6.53) | 5.56 (5.11, 6.06) | 5.78 (5.32, 6.28) |
| Betaine (N,N,N-trimethylglycine) | SAAR | 28.1 (25, 31.6) | 29.5 (25.9, 33.7) | 32.8 (28.8, 37.5) |
|  | Control | 28.1 (25, 31.6) | 30.2 (26.4, 34.6) | 31.7 (27.7, 36.1) |
| N,N-dimethylglycine | SAAR | 3.58 (3.29, 3.89) | 3.5 (3.18, 3.86) | 3.8 (3.45, 4.19) |
|  | Control | 3.58 (3.29, 3.89) | 3.51 (3.18, 3.88) | 3.66 (3.32, 4.03) |
| Glycine | SAAR | 99.1 (93.7, 105) | 114 (107, 122) | 109 (102, 117) |
|  | Control | 99.1 (93.7, 105) | 109 (102, 117) | 107 (100, 114) |
| Serine | SAAR | 210 (192, 230) | 242 (219, 267) | 245 (222, 271) |
|  | Control | 210 (192, 230) | 240 (216, 266) | 247 (224, 274) |
| Total glutathione | SAAR | 6.45 (6.02, 6.91) | 6.47 (5.97, 7.02) | 6.63 (6.11, 7.2) |
|  | Control | 6.45 (6.02, 6.91) | 6.48 (5.95, 7.05) | 6.18 (5.69, 6.71) |
| Total cysteinylglycine | SAAR | 25.1 (23.6, 26.6) | 24.7 (23.1, 26.4) | 24.6 (23, 26.3) |
|  | Control | 25.1 (23.6, 26.6) | 23.9 (22.3, 25.6) | 23.8 (22.2, 25.5) |
| Total γ-glutamylcysteine | SAAR | 3.96 (3.76, 4.17) | 4.12 (3.88, 4.38) | 4.1 (3.86, 4.36) |
|  | Control | 3.96 (3.76, 4.17) | 3.94 (3.7, 4.2) | 3.90 (3.67, 4.15) |
| Hypotaurine | SAAR | 0.72 (0.65, 0.80) | 0.62 (0.54, 0.70) | 0.64 (0.56, 0.72) |
|  | Control | 0.72 (0.65, 0.80) | 0.95 (0.83, 1.08) | 1.00 (0.88, 1.14) |
| Taurine | SAAR | 56.6 (53, 60.5) | 52.3 (47.9, 57.2) | 53.7 (49.0, 58.7) |
|  | Control | 56.6 (53, 60.5) | 53.1 (48.3, 58.3) | 55.4 (50.6, 60.6) |
| Sulfide, bioavailable | SAAR | 0.05 (0.045, 0.055) | 0.046 (0.04, 0.053) | 0.039 (0.034, 0.045) |
|  | Control | 0.05 (0.045, 0.055) | 0.054 (0.047, 0.063) | 0.049 (0.043, 0.057) |
| Homolanthionine | SAAR | 0.008 (0.007, 0.01) | 0.005 (0.004, 0.007) | 0.005 (0.004, 0.006) |
|  | Control | 0.008 (0.007, 0.01) | 0.011 (0.009, 0.013) | 0.012 (0.01, 0.015) |
| Lanthionine | SAAR | 0.055 (0.05, 0.06) | 0.049 (0.045, 0.055) | 0.05 (0.048, 0.06) |
|  | Control | 0.055 (0.05, 0.06) | 0.058 (0.052, 0.064) | 0.061 (0.055, 0.068) |
| Sulfite | SAAR | 0.055 (0.044, 0.069) | 0.051 (0.040, 0.065) | 0.048 (0.038, 0.062) |
|  | Control | 0.055 (0.044, 0.069) | 0.077 (0.060, 0.097) | 0.088 (0.069, 0.112) |
| S-sulfocysteine | SAAR | 0.14 (0.13, 0.16) | 0.13 (0.11, 0.15) | 0.14 (0.12, 0.16) |
|  | Control | 0.14 (0.13, 0.16) | 0.26 (0.21, 0.31) | 0.30 (0.26, 0.36) |
| Thiosulfate | SAAR | 0.3 (0.28, 0.33) | 0.26 (0.23, 0.29) | 0.26 (0.23, 0.30) |
|  | Control | 0.3 (0.28, 0.33) | 0.46 (0.41, 0.53) | 0.51 (0.45, 0.57) |
| Pyruvate | SAAR | 29.4 (26.2, 32.9) | 27.2 (23.4, 31.8) | 25.1 (21.5, 29.4) |
|  | Control | 29.4 (26.2, 32.9) | 23 (19.6, 27) | 27.7 (23.7, 32.4) |
| Alanine | SAAR | 307 (290, 325) | 294 (274, 316) | 281 (262, 302) |
|  | Control | 307 (290, 325) | 296 (275, 319) | 292 (272, 314) |
| Glutamate | SAAR | 31.7 (27.1, 37.0) | 30.1 (24.7, 36.5) | 25.1 (20.6, 30.6) |
|  | Control | 31.7 (27.1, 37.0) | 29.0 (23.6, 35.7) | 28.5 (23.4, 34.8) |
| Glutamine | SAAR | 399 (378, 421) | 436 (410, 464) | 422 (396, 448) |
|  | Control | 399 (378, 421) | 413 (388, 440) | 406 (381, 431) |
| Histidine | SAAR | 71.1 (68.7, 73.6) | 71.5 (68.3, 74.9) | 72.9 (69.6, 76.3) |
|  | Control | 71.1 (68.7, 73.6) | 72 (68.7, 75.6) | 68 (64.9, 71.3) |
| ^1^All values are estimated marginal geometric means derived from linear mixed regression models log-transformed metabolite as the outcome, and group, visit and their interaction term (group × time) at 4 and 8 weeks as predictors. The models were baseline adjusted. Subject ID was added as a random variable to account for within-subject correlation. Abbreviations: SAAR, sulfur amino acid restriction | | | | |

| **Appendix Table A.6.** Estimated marginal means of 24-h urine sulfur amino acids (μmol/24 h) and related metabolites | | | | |
| --- | --- | --- | --- | --- |
|  |  | Baseline | 4 weeks | 8 weeks |
| Methionine | SAAR | 8.89 (7.57, 10.4) | 5.27 (4.29, 6.47) | 5.85 (4.74, 7.21) |
|  | Control | 8.89 (7.57, 10.4) | 9.26 (7.47, 11.5) | 9.24 (7.43, 11.5) |
| Sarcosine (N-methylglycine) | SAAR | 1.93 (1.63, 2.29) | 1.42 (1.15, 1.75) | 1.58 (1.28, 1.96) |
|  | Control | 1.93 (1.63, 2.29) | 1.42 (1.14, 1.77) | 1.4 (1.12, 1.75) |
| Total homocysteine | SAAR | 8.19 (7.16, 9.37) | 5.8 (4.84, 6.96) | 5.54 (4.6, 6.66) |
|  | Control | 8.19 (7.16, 9.37) | 8.64 (7.13, 10.5) | 8.13 (6.69, 9.89) |
| Cystathionine | SAAR | 33.5 (26.8, 42) | 6.40 (4.73, 8.66) | 6.90 (5.06, 9.39) |
|  | Control | 33.5 (26.8, 42) | 28.2 (20.5, 38.7) | 32.9 (23.7, 45.5) |
| Total cysteine | SAAR | 243 (219, 271) | 235 (203, 272) | 214 (184, 248) |
|  | Control | 243 (219, 271) | 301 (258, 352) | 283 (242, 332) |
| Choline | SAAR | 24.2 (21.4, 27.3) | 17.7 (15, 20.8) | 18.2 (15.4, 21.6) |
|  | Control | 24.2 (21.4, 27.3) | 21.2 (17.8, 25.2) | 19.9 (16.7, 23.8) |
| Betaine (N,N,N-trimethylglycine) | SAAR | 59.6 (49.3, 72) | 36.7 (28.9, 46.6) | 43.0 (33.8, 54.8) |
|  | Control | 59.6 (49.3, 72) | 38.5 (30, 49.4) | 41.7 (32.4, 53.7) |
| N,N-dimethylglycine | SAAR | 57.8 (47.1, 70.8) | 37.5 (29.4, 47.8) | 41.7 (32.6, 53.3) |
|  | Control | 57.8 (47.1, 70.8) | 40.1 (31.2, 51.6) | 41.0 (31.8, 52.9) |
| Glycine | SAAR | 1640 (1420, 1900) | 1410 (1170, 1700) | 1530 (1260, 1840) |
|  | Control | 1640 (1420, 1900) | 1350 (1110, 1640) | 1350 (1110, 1650) |
| Serine | SAAR | 414 (360, 476) | 382 (319, 456) | 405 (338, 485) |
|  | Control | 414 (360, 476) | 391 (325, 472) | 345 (286, 417) |
| Total glutathione | SAAR | 5.80 (4.92, 6.83) | 6.41 (5.22, 7.87) | 5.42 (4.39, 6.69) |
|  | Control | 5.80 (4.92, 6.83) | 4.48 (3.68, 5.46) | 4.70 (3.85, 5.74) |
| Total cysteinylglycine | SAAR | 7.95 (6.92, 9.12) | 5.67 (4.7, 6.84) | 5.67 (4.68, 6.86) |
|  | Control | 7.95 (6.92, 9.12) | 8.10 (6.64, 9.86) | 7.54 (6.16, 9.23) |
| Taurine | SAAR | 425 (310, 585) | 232 (153, 351) | 321 (210, 489) |
|  | Control | 425 (310, 585) | 722 (468, 1110) | 752 (483, 1170) |
| Homolanthionine | SAAR | 1.24 (1.06, 1.46) | 0.842 (0.676, 1.05) | 0.957 (0.765, 1.20) |
|  | Control | 1.24 (1.06, 1.46) | 2.23 (1.77, 2.81) | 2.09 (1.65, 2.64) |
| Lanthionine | SAAR | 15 (13.2, 16.9) | 13.2 (11.1, 15.6) | 14.3 (12.1, 17) |
|  | Control | 15 (13.2, 16.9) | 17.7 (14.8, 21.1) | 14.6 (12.2, 17.5) |
| Sulfite | SAAR | 1.97 (1.35, 2.87) | 2.11 (1.31, 3.39) | 2.51 (1.52, 4.15) |
|  | Control | 1.97 (1.35, 2.87) | 2.74 (1.71, 4.39) | 4.09 (2.51, 6.65) |
| S-sulfocysteine | SAAR | 6.14 (5.29, 7.13) | 4.35 (3.55, 5.33) | 4.65 (3.78, 5.72) |
|  | Control | 6.14 (5.29, 7.13) | 16.0 (12.9, 19.8) | 14.9 (11.9, 18.5) |
| Thiosulfate | SAAR | 15.9 (12.9, 19.5) | 7.56 (5.68, 10) | 7.58 (5.67, 10.1) |
|  | Control | 15.9 (12.9, 19.5) | 71.4 (52.9, 96.4) | 83.0 (61, 113) |
| Sulfate | SAAR | 17100 (14800, 19700) | 6580 (5400, 8010) | 6690 (5470, 8180) |
|  | Control | 17100 (14800, 19700) | 28100 (22800, 34600) | 26800 (21700, 33200) |
| Alanine | SAAR | 318 (276, 368) | 194 (160, 234) | 209 (172, 253) |
|  | Control | 318 (276, 368) | 187 (153, 228) | 180 (147, 221) |
| Glutamate | SAAR | 29.1 (25.6, 33.2) | 29 (24.3, 34.6) | 29.9 (25, 35.7) |
|  | Control | 29.1 (25.6, 33.2) | 23.7 (19.7, 28.5) | 22.3 (18.4, 26.9) |
| Glutamine | SAAR | 509 (446, 581) | 405 (340, 481) | 423 (355, 504) |
|  | Control | 509 (446, 581) | 366 (306, 439) | 335 (279, 403) |
| ^1^All values are estimated marginal geometric means derived from linear mixed regression models log-transformed metabolite as the outcome, and group, visit and their interaction term (group × time) at 4 and 8 weeks as predictors. The models were baseline adjusted. Subject ID was added as a random variable to account for within-subject correlation. Abbreviations: SAAR, sulfur amino acid restriction | | | | |

| **Appendix Table A.7.** Estimated marginal mean excretional fraction of sulfur amino acids (%) and related metabolites | | | | |
| --- | --- | --- | --- | --- |
|  |  | Baseline | 4 weeks | 8 weeks |
| Methionine | SAAR | 0.234 (0.213, 0.256) | 0.175 (0.154, 0.198) | 0.19 (0.167, 0.216) |
|  | Control | 0.234 (0.213, 0.256) | 0.197 (0.172, 0.225) | 0.231 (0.202, 0.264) |
| Sarcosine (N-methylglycine) | SAAR | 0.679 (0.567, 0.812) | 0.543 (0.434, 0.68) | 0.618 (0.493, 0.775) |
|  | Control | 0.679 (0.567, 0.812) | 0.421 (0.329, 0.538) | 0.421 (0.333, 0.533) |
| Cystathionine | SAAR | 73.8 (60.4, 90.3) | 29.6 (22.4, 39) | 26.6 (20, 35.3) |
|  | Control | 73.8 (60.4, 90.3) | 39.4 (29.2, 53.1) | 49.9 (36.9, 67.3) |
| Choline | SAAR | 1.75 (1.59, 1.92) | 1.50 (1.33, 1.70) | 1.47 (1.30, 1.66) |
|  | Control | 1.75 (1.59, 1.92) | 1.71 (1.50, 1.95) | 1.78 (1.56, 2.02) |
| N,N,N-trimethylglycine (betaine) | SAAR | 0.852 (0.702, 1.03) | 0.498 (0.39, 0.637) | 0.506 (0.395, 0.65) |
|  | Control | 0.852 (0.702, 1.03) | 0.499 (0.385, 0.648) | 0.655 (0.506, 0.848) |
| N,N-dimethylglycine | SAAR | 6.66 (5.45, 8.12) | 4.28 (3.38, 5.44) | 4.49 (3.53, 5.71) |
|  | Control | 6.66 (5.45, 8.12) | 4.73 (3.68, 6.07) | 5.23 (4.08, 6.7) |
| Glycine | SAAR | 3.23 (2.82, 3.69) | 2.53 (2.14, 3) | 2.54 (2.14, 3.02) |
|  | Control | 3.23 (2.82, 3.69) | 2.17 (1.82, 2.6) | 2.49 (2.09, 2.98) |
| Serine | SAAR | 2.02 (1.82, 2.25) | 1.68 (1.47, 1.92) | 1.86 (1.63, 2.13) |
|  | Control | 2.02 (1.82, 2.25) | 1.74 (1.51, 2) | 1.82 (1.58, 2.08) |
| Taurine | SAAR | 2.75 (1.98, 3.83) | 1.35 (0.89, 2.06) | 1.72 (1.12, 2.63) |
|  | Control | 2.75 (1.98, 3.83) | 4.67 (3.00, 7.27) | 5.03 (3.24, 7.81) |
| Homolanthionine | SAAR | 77.9 (68.8, 88.3) | 79.1 (66.5, 94.1) | 94.5 (79.2, 113) |
|  | Control | 77.9 (68.8, 88.3) | 113 (93.7, 136) | 90.8 (75.5, 109) |
| Lanthionine | SAAR | 128 (113, 144) | 137 (117, 160) | 136 (116, 159) |
|  | Control | 128 (113, 144) | 153 (130, 181) | 138 (118, 163) |
| S-sulfocysteine | SAAR | 17.6 (14.5, 21.4) | 13.8 (10.6, 17.9) | 13.2 (10.2, 17.2) |
|  | Control | 17.6 (14.5, 21.4) | 27.2 (20.1, 36.9) | 20.6 (15.4, 27.6) |
| Thiosulfate | SAAR | 25.4 (21.4, 30.1) | 14.1 (11.1, 17.9) | 13.7 (10.7, 17.5) |
|  | Control | 25.4 (21.4, 30.1) | 67.0 (51.8, 86.7) | 52.1 (40.4, 67.3) |
| Alanine | SAAR | 0.429 (0.372, 0.495) | 0.27 (0.225, 0.324) | 0.31 (0.258, 0.372) |
|  | Control | 0.429 (0.372, 0.495) | 0.27 (0.223, 0.327) | 0.307 (0.253, 0.371) |
| Glutamate | SAAR | 0.407 (0.339, 0.488) | 0.43 (0.342, 0.541) | 0.54 (0.428, 0.682) |
|  | Control | 0.407 (0.339, 0.488) | 0.357 (0.28, 0.455) | 0.427 (0.335, 0.543) |
| Glutamine | SAAR | 0.583 (0.519, 0.656) | 0.459 (0.399, 0.529) | 0.486 (0.421, 0.56) |
|  | Control | 0.583 (0.519, 0.656) | 0.428 (0.369, 0.496) | 0.493 (0.426, 0.572) |
| ^1^All values are estimated marginal geometric means derived from linear mixed regression models log-transformed metabolite as the outcome, and group, visit and their interaction term (group × time) at 4 and 8 weeks as predictors. The models were baseline adjusted. Subject ID was added as a random variable to account for within-subject correlation. Abbreviations: SAAR, sulfur amino acid restriction | | | | |

| **Appendix Table A.8.** Within-group changes vs. baseline (%) for plasma, urine and excretional fraction of metabolites in the sulfur amino acid restriction group^1^ | | | | |
| --- | --- | --- | --- | --- |
|  | **4 weeks** | | **8 weeks** | |
|  | **β (95 % CI)** | **p** | **β (95 % CI)** | **p** |
| ***Plasma*** |  |  |  |  |
| Methionine | -3.1 (-8.54, 2.66) | 0.28 | -4.54 (-9.97, 1.21) | 0.12 |
| S-adenosylmethionine | 2.66 (-1.52, 7.02) | 0.21 | 1.76 (-2.31, 6) | 0.4 |
| Sarcosine (N-methylglycine) | -26.5 (-34.1, -17.9) | < 0.001 | -25.5 (-33.2, -16.8) | < 0.001 |
| S-adenosylhomocysteine | -15.5 (-26, -3.65) | 0.013 | -15.8 (-26, -4.15) | 0.01 |
| Total homocysteine | 4.18 (-1.94, 10.7) | 0.18 | 7.76 (1.36, 14.6) | 0.018 |
| Cystathionine | -48 (-55.9, -38.6) | < 0.001 | -45.3 (-53.7, -35.4) | < 0.001 |
| Total cysteine | 1.92 (-1.97, 5.97) | 0.33 | 3.63 (-0.377, 7.79) | 0.075 |
| Choline | -14.7 (-21.7, -7.2) | < 0.001 | -10.2 (-17.6, -2.17) | 0.015 |
| Betaine (N,N,N-trimethylglycine) | 5.3 (-3.22, 14.6) | 0.23 | 17.1 (7.49, 27.5) | < 0.001 |
| N,N-dimethylglycine | -2.11 (-8.31, 4.51) | 0.52 | 6.15 (-0.668, 13.4) | 0.077 |
| Glycine | 15.4 (8.72, 22.5) | < 0.001 | 17.2 (10.4, 24.6) | < 0.001 |
| Serine | 15.7 (10.2, 21.4) | < 0.001 | 10.8 (5.5, 16.3) | < 0.001 |
| Total glutathione | 0.521 (-5.19, 6.57) | 0.86 | 3.01 (-2.91, 9.3) | 0.32 |
| Total cysteinylglycine | -1.52 (-5.41, 2.53) | 0.45 | -1.98 (-5.9, 2.11) | 0.33 |
| Total γ-glutamylcysteine | 4.62 (-0.0179, 9.47) | 0.051 | 4.09 (-0.585, 8.98) | 0.086 |
| Hypotaurine | -15.5 (-24.8, -5.16) | 0.005 | -12.8 (-22.5, -1.96) | 0.023 |
| Taurine | -7.74 (-16.2, 1.63) | 0.1 | -5.45 (-14.3, 4.28) | 0.26 |
| Sulfide, bioavailable | -9.67 (-23.9, 7.22) | 0.24 | -24.3 (-36.4, -9.98) | 0.002 |
| Homolanthionine | -36.8 (-47.6, -23.6) | < 0.001 | -39.7 (-50.2, -27) | < 0.001 |
| Lanthionine | -9.1 (-16.5, -0.988) | 0.029 | -1.76 (-9.91, 7.12) | 0.68 |
| Sulfite | -8.06 (-13, -2.82) | 0.004 | -12.4 (-17.2, -7.3) | < 0.001 |
| S-sulfocysteine | -8.36 (-19.3, 4.08) | 0.17 | 0.994 (-11.1, 14.7) | 0.88 |
| Thiosulfate | -14.3 (-21, -6.98) | < 0.001 | -11.7 (-18.6, -4.07) | 0.004 |
| Pyruvate | -6.39 (-20.1, 9.7) | 0.41 | -13.5 (-26.4, 1.52) | 0.075 |
| Alanine | -2.6 (-8.29, 3.45) | 0.38 | -6.82 (-12.3, -0.961) | 0.024 |
| Glutamate | -3.01 (-20.5, 18.4) | 0.76 | -19.2 (-33.9, -1.12) | 0.039 |
| Glutamine | 9.66 (5.43, 14.1) | < 0.001 | 5.97 (1.82, 10.3) | 0.005 |
| Histidine | 1.41 (-2.94, 5.96) | 0.52 | 3.23 (-1.19, 7.86) | 0.15 |
| ***Urine*** |  |  |  |  |
| Methionine | -43.4 (-53.1, -31.8) | < 0.001 | -37.1 (-48, -23.9) | < 0.001 |
| Sarcosine (N-methylglycine) | -25.8 (-39.2, -9.29) | 0.004 | -17.1 (-32.3, 1.58) | 0.07 |
| Total homocysteine | -33.6 (-44.9, -19.9) | < 0.001 | -36.6 (-47.5, -23.4) | < 0.001 |
| Cystathionine | -82.9 (-87.4, -76.7) | < 0.001 | -81.4 (-86.4, -74.7) | < 0.001 |
| Total cysteine | -7.29 (-22.6, 11.1) | 0.41 | -15.9 (-29.9, 1) | 0.063 |
| Choline | -27.8 (-41, -11.6) | 0.002 | -25.8 (-39.5, -9.01) | 0.005 |
| Betaine (N,N,N-trimethylglycine) | -39.2 (-51.4, -23.9) | < 0.001 | -28.6 (-43.1, -10.4) | 0.004 |
| N,N-dimethylglycine | -36.3 (-48.3, -21.4) | < 0.001 | -29.2 (-42.7, -12.4) | 0.002 |
| Glycine | -14 (-27.6, 2.24) | 0.086 | -6.51 (-21.5, 11.4) | 0.44 |
| Serine | -10.7 (-26, 7.71) | 0.23 | -5.31 (-21.7, 14.5) | 0.57 |
| Taurine | -41.5 (-60.2, -14.2) | 0.007 | -19.8 (-45.6, 18.4) | 0.26 |
| Total glutathione | -24 (-36.8, -8.52) | 0.004 | -20.4 (-34, -4.01) | 0.018 |
| Total cysteinylglycine | -30.4 (-45.2, -11.6) | 0.004 | -31.1 (-45.9, -12.3) | 0.003 |
| Homolanthionine | -36.9 (-49.6, -20.9) | < 0.001 | -27.5 (-42.3, -8.87) | 0.007 |
| Lanthionine | -14.8 (-31.5, 5.87) | 0.14 | -7.38 (-25.7, 15.4) | 0.49 |
| Sulfite | 6.76 (-27.4, 57) | 0.73 | 26.5 (-16.5, 91.6) | 0.25 |
| S-sulfocysteine | -32.2 (-45.2, -16.2) | < 0.001 | -27.5 (-41.5, -10.2) | 0.004 |
| Thiosulfate | -55.6 (-65.4, -43.1) | < 0.001 | -55.4 (-65.3, -42.7) | < 0.001 |
| Sulfate | -62.9 (-71.3, -51.9) | < 0.001 | -62.3 (-71, -51.1) | < 0.001 |
| Glutamate | -0.002 (-18.1, 22) | 1 | 3.36 (-15.5, 26.5) | 0.74 |
| Glutamine | -23.7 (-37.2, -7.45) | 0.007 | -20.3 (-34.5, -3.07) | 0.024 |
| Alanine | -40.8 (-52, -27.1) | < 0.001 | -36.1 (-48.3, -21.1) | < 0.001 |
| ***Excretional fraction*** |  |  |  |  |
| Methionine | -26.2 (-35.3, -15.7) | < 0.001 | -19.8 (-29.8, -8.34) | 0.002 |
| Sarcosine (N-methylglycine) | -21.7 (-36, -4.26) | 0.018 | -10.8 (-26.9, 8.93) | 0.26 |
| Cystathionine | -60.4 (-70.6, -46.8) | < 0.001 | -64.3 (-73.5, -51.8) | < 0.001 |
| Choline | -13.2 (-24.3, -0.427) | 0.044 | -15.2 (-26.2, -2.56) | 0.021 |
| Betaine (N,N,N-trimethylglycine) | -41.2 (-54.6, -23.9) | < 0.001 | -40.5 (-54.2, -22.7) | < 0.001 |
| N,N-dimethylglycine | -36.9 (-48.8, -22.3) | < 0.001 | -33.8 (-46.4, -18.3) | < 0.001 |
| Glycine | -21.5 (-32.1, -9.29) | 0.001 | -21.2 (-32, -8.82) | 0.002 |
| Serine | -18.4 (-28.6, -6.78) | 0.003 | -9.47 (-20.9, 3.6) | 0.15 |
| Taurine | -47.5 (-65.2, -20.7) | 0.003 | -33.4 (-56.1, 1.08) | 0.056 |
| Homolanthionine | -2.5 (-21.3, 20.9) | 0.81 | 16.5 (-6.25, 44.7) | 0.16 |
| Lanthionine | 5.39 (-9.27, 22.4) | 0.49 | 4.3 (-10.4, 21.4) | 0.58 |
| S-sulfocysteine | -22.3 (-40.1, 0.928) | 0.058 | -25.3 (-42.4, -3) | 0.03 |
| Thiosulfate | -45.7 (-55.6, -33.7) | < 0.001 | -47.9 (-57.5, -36.2) | < 0.001 |
| ^1^All values are back-transformed β-estimates with corresponding confidence intervals estimated marginal derived from linear mixed regression models with log-transformed metabolite as the outcome, and group, visit at 4 and 8 weeks as predictors. Subject ID was added as a random variable to account for within-subject correlation. Abbreviations: SAAR, sulfur amino acid restriction | | | | |

| **Appendix Table A.9.** Within-group changes vs. baseline (%) for plasma, urine and excretional fraction of metabolites in the control group | | | | |
| --- | --- | --- | --- | --- |
|  | **4 weeks** | | **8 weeks** | |
|  | **β (95 % CI)** | **p** | **β (95 % CI)** | **p** |
| ***Plasma*** |  |  |  |  |
| Methionine | -2.62 (-9.31, 4.57) | 0.46 | -1.04 (-7.61, 6) | 0.76 |
| S-adenosylmethionine | 3.25 (-1.19, 7.89) | 0.15 | 3.19 (-1.12, 7.69) | 0.15 |
| Sarcosine (N-methylglycine) | 1.87 (-9.39, 14.5) | 0.75 | 10.7 (-1.15, 23.9) | 0.077 |
| S-adenosylhomocysteine | -14.5 (-23.1, -5.01) | 0.004 | -14 (-22.4, -4.7) | 0.005 |
| Total homocysteine | -15.1 (-19.9, -10.1) | < 0.001 | -13.1 (-17.8, -8.1) | < 0.001 |
| Cystathionine | 11 (-13.4, 42.2) | 0.4 | 21.5 (-4.46, 54.4) | 0.11 |
| Total cysteine | -4.37 (-8.28, -0.28) | 0.037 | -2.43 (-6.28, 1.59) | 0.23 |
| Choline | -10.3 (-18.1, -1.7) | 0.021 | -6.77 (-14.7, 1.85) | 0.12 |
| Betaine (N,N,N-trimethylglycine) | 7.08 (-2.14, 17.2) | 0.13 | 12.2 (2.92, 22.4) | 0.01 |
| N,N-dimethylglycine | -1.89 (-9.53, 6.4) | 0.64 | 2.3 (-5.4, 10.6) | 0.56 |
| Glycine | 13.5 (6.77, 20.6) | < 0.001 | 17 (10.4, 24.1) | < 0.001 |
| Serine | 9.4 (3.33, 15.8) | 0.003 | 7.61 (1.84, 13.7) | 0.01 |
| Total glutathione | 0.217 (-6.92, 7.9) | 0.95 | -4.37 (-11, 2.69) | 0.21 |
| Total cysteinylglycine | -4.59 (-9.01, 0.0427) | 0.052 | -4.95 (-9.2, -0.513) | 0.03 |
| Total γ-glutamylcysteine | -1.18 (-6.3, 4.22) | 0.66 | -2.08 (-6.97, 3.07) | 0.41 |
| Hypotaurine | 33.3 (20, 48.1) | < 0.001 | 41.1 (27.5, 56.2) | < 0.001 |
| Taurine | -5.85 (-15, 4.26) | 0.24 | -1.6 (-10.8, 8.61) | 0.74 |
| Sulfide, bioavailable | 12.4 (-1.97, 28.9) | 0.092 | 1.59 (-11, 15.9) | 0.81 |
| Homolanthionine | 30.6 (3.19, 65.3) | 0.027 | 47.3 (17.3, 84.9) | 0.001 |
| Lanthionine | 4.89 (-3.55, 14.1) | 0.26 | 11.2 (2.53, 20.5) | 0.011 |
| Sulfite | 39.3 (17, 65.9) | < 0.001 | 59.8 (34.6, 89.7) | < 0.001 |
| S-sulfocysteine | 72.9 (31, 128) | < 0.001 | 105 (57.1, 168) | < 0.001 |
| Thiosulfate | 51.9 (23.7, 86.6) | < 0.001 | 65.5 (35.6, 102) | < 0.001 |
| Pyruvate | -22.5 (-35.8, -6.57) | 0.009 | -6.81 (-22.2, 11.7) | 0.44 |
| Alanine | -5.42 (-12.2, 1.9) | 0.14 | -6.71 (-13.2, 0.251) | 0.058 |
| Glutamate | -12.1 (-27, 5.76) | 0.17 | -13.2 (-27.4, 3.77) | 0.12 |
| Glutamine | 3.25 (-1.55, 8.28) | 0.18 | 1.31 (-3.23, 6.06) | 0.57 |
| Histidine | 0.119 (-5.48, 6.05) | 0.97 | -5.44 (-10.5, -0.0432) | 0.048 |
| ***Urine*** |  |  |  |  |
| Methionine | 8.91 (-13, 36.3) | 0.45 | 8.43 (-13.6, 36.1) | 0.48 |
| Sarcosine (N-methylglycine) | -27.2 (-40.3, -11.2) | 0.002 | -28.2 (-41.3, -12.2) | 0.002 |
| Total homocysteine | 13 (-10.2, 42.1) | 0.29 | 6.23 (-15.8, 34) | 0.6 |
| Cystathionine | -6.91 (-36.9, 37.4) | 0.71 | 8.22 (-27.1, 60.6) | 0.69 |
| Total cysteine | 29.7 (10.2, 52.6) | 0.002 | 22.4 (3.8, 44.4) | 0.017 |
| Choline | -10.7 (-24.7, 5.9) | 0.19 | -16.1 (-29.5, -0.287) | 0.046 |
| Betaine (N,N,N-trimethylglycine) | -35 (-49.4, -16.5) | 0.001 | -28.8 (-44.8, -8.24) | 0.01 |
| N,N-dimethylglycine | -29.2 (-42.1, -13.5) | 0.001 | -27.7 (-41, -11.3) | 0.002 |
| Glycine | -18.7 (-32.7, -1.63) | 0.034 | -18.3 (-32.7, -0.981) | 0.04 |
| Serine | -2.44 (-17.7, 15.6) | 0.77 | -14 (-27.6, 2.16) | 0.085 |
| Taurine | 54.4 (-5.6, 153) | 0.082 | 65.3 (0.421, 172) | 0.048 |
| Total glutathione | 12.7 (-3.31, 31.4) | 0.12 | -4.92 (-18.8, 11.4) | 0.52 |
| Total cysteinylglycine | 5.86 (-12.8, 28.5) | 0.56 | -1.36 (-19, 20.1) | 0.89 |
| Homolanthionine | 93 (44.6, 158) | < 0.001 | 79.9 (34.3, 141) | < 0.001 |
| Lanthionine | 22.5 (2.4, 46.4) | 0.027 | 1.26 (-15.5, 21.4) | 0.89 |
| Sulfite | 40.4 (-7.64, 113) | 0.11 | 108 (34.8, 222) | 0.002 |
| S-sulfocysteine | 172 (108, 256) | < 0.001 | 153 (92.7, 231) | < 0.001 |
| Thiosulfate | 382 (211, 645) | < 0.001 | 458 (259, 768) | < 0.001 |
| Sulfate | 71.4 (34.1, 119) | < 0.001 | 63.5 (27.5, 110) | < 0.001 |
| Glutamate | -19.5 (-33.7, -2.18) | 0.03 | -24.6 (-38.1, -8.16) | 0.006 |
| Glutamine | -24.8 (-36.1, -11.5) | < 0.001 | -31.1 (-41.6, -18.8) | < 0.001 |
| Alanine | -40.1 (-51, -26.7) | < 0.001 | -42.6 (-53.2, -29.6) | < 0.001 |
| ***FE*** |  |  |  |  |
| Methionine | -15.2 (-28.9, 0.997) | 0.064 | -0.883 (-16.7, 17.9) | 0.92 |
| Sarcosine (N-methylglycine) | -37.1 (-50.8, -19.5) | < 0.001 | -37.1 (-50.3, -20.4) | < 0.001 |
| Cystathionine | -46.3 (-64, -19.7) | 0.003 | -32.4 (-54.8, 0.964) | 0.056 |
| Choline | -3.27 (-15.1, 10.3) | 0.61 | 0.511 (-11.7, 14.4) | 0.94 |
| Betaine (N,N,N-trimethylglycine) | -42.7 (-54.6, -27.7) | < 0.001 | -24.1 (-39.7, -4.5) | 0.02 |
| N,N-dimethylglycine | -27.7 (-40.6, -12.1) | 0.002 | -20.3 (-34.3, -3.23) | 0.023 |
| Glycine | -34.1 (-46, -19.6) | < 0.001 | -24.1 (-37.7, -7.67) | 0.007 |
| Serine | -11.4 (-20.4, -1.47) | 0.026 | -7.74 (-16.9, 2.47) | 0.13 |
| Taurine | 56 (2.1, 138) | 0.04 | 67.9 (10.6, 155) | 0.016 |
| Homolanthionine | 52 (20.1, 92.3) | < 0.001 | 22.3 (-3.1, 54.4) | 0.089 |
| Lanthionine | 22.5 (3.72, 44.7) | 0.018 | 10.4 (-6.3, 30.1) | 0.23 |
| S-sulfocysteine | 56.7 (2.16, 140) | 0.04 | 18.8 (-21.7, 80.4) | 0.4 |
| Thiosulfate | 171 (79.7, 309) | < 0.001 | 110 (39.5, 215) | < 0.001 |

| **Appendix Table A.10.** Unadjusted geometric mean (geometric standard deviation) concentrations of other amino acids and intermediates at baseline^1^ | | | | | | |
| --- | --- | --- | --- | --- | --- | --- |
|  | ***Plasma, μmol/L*** | | ***24-h urine, μmol/24 h*** | | ***Excretional fraction, %*** | |
|  | **SAAR** | **Controls** | **SAAR** | **Controls** | **SAAR** | **Controls** |
| Pyruvate | 29.4 (1.51) | 29.8 (1.44) | - | - | - | - |
| Alanine | 295 (1.21) | 327 (1.21) | 307 (1.83) | 281 (1.76) | 0.46 (1.64) | 0.40 (1.71) |
| Glutamate | 28.3 (1.68) | 31.3 (1.88) | 28.2 (1.53) | 28.7 (1.53) | 0.47 (1.80) | 0.43 (2.05) |
| Glutamine | 380 (1.16) | 402 (1.23) | 505 (1.77) | 430 (1.67) | 0.62 (1.54) | 0.53 (1.62) |
| Histidine | 69.6 (1.13) | 72.2 (1.14) | - | - | - | - |
| ^1^All values are geometric mean (geometric standard deviations) | | | | | | |

| **Appendix Table A.11. β-estimates (95 % and confidence intervals) of other amino acids in plasma and urine, and excretional fraction vs. controls^1^** | | | | | |
| --- | --- | --- | --- | --- | --- |
|  | **4 weeks** | | **8 weeks** | | |
|  | **β (95 % CI)** | **p** | **β (95 % CI)** | **p** | **p_adjusted_** |
| ***Plasma*** | | | | | |
| Pyruvate | 18.5 (-3.18, 45) | 0.099 | -9.13 (-25.6, 11) | 0.35 | 0.48 |
| Alanine | -0.724 (-8.79, 8.06) | 0.87 | -3.76 (-11.5, 4.62) | 0.37 | 0.49 |
| Glutamate | 3.6 (-18.7, 32) | 0.77 | -11.9 (-30.6, 11.8) | 0.3 | 0.43 |
| Glutamine | 5.57 (-0.365, 11.9) | 0.066 | 3.96 (-1.81, 10.1) | 0.18 | 0.31 |
| Histidine | -0.738 (-6.49, 5.37) | 0.81 | 7.13 (1.03, 13.6) | 0.022 | 0.053 |
| ***Urine*** | | | | | |
| Glutamate | 22.1 (-3.1, 53.9) | 0.09 | 34.2 (6.01, 69.8) | 0.015 | 0.041 |
| Glutamine | 10.4 (-11.3, 37.5) | 0.37 | 26.1 (0.891, 57.7) | 0.042 | 0.092 |
| Alanine | 3.62 (-18.9, 32.4) | 0.77 | 15.7 (-9.85, 48.4) | 0.25 | 0.40 |
| ***Excretional fraction*** | | | | | |
| Glutamate | 22.1 (-3.1, 53.9) | 0.09 | 34.2 (6.01, 69.8) | 0.015 | 0.041 |
| Glutamine | 10.4 (-11.3, 37.5) | 0.37 | 26.1 (0.891, 57.7) | 0.042 | 0.092 |
| Alanine | 3.62 (-18.9, 32.4) | 0.77 | 15.7 (-9.85, 48.4) | 0.25 | 0.40 |
| ^1^Values are derived from linear mixed regression models with log-transformed metabolite as the outcome, and group, visit and their interaction term (group × time) at 4 and 8 weeks as predictors. The models were baseline adjusted. Subject ID was added as a random term to account for within-subject correlation. All estimates are back-transformed and represent % difference vs controls. P-values were derived from the regression models. Adjusted 8-week p-value data were calculated using the Benjamini-Hochberg procedure. | | | | | |
|  |  |  |  |  |  |

| **Appendix Table A.12.** Correlation coefficients (Spearman’s rho) for principal component 1 and change body mass outcomes | | |
| --- | --- | --- |
|  | SAAR | Control |
| Body weight change | rho = -0.56  p = 0.003 | rho = 0.09  p = 0.67 |
| Total fat mass change | rho = -0.52  p = 0.006 | rho = 0.06  p = 0.77 |
| Android fat mass change | rho = -0.44  p = 0.02 | rho = 0.15  p = 0.47 |
| Gynoid fat mass change | rho = -0.50  p = 0.008 | rho = 0.00  p = 0.98 |
